# Depth-Dependent Environmental Drivers of Microbial Plankton Community Structure in the Northern Gulf of Mexico

**DOI:** 10.3389/fmicb.2018.03175

**Published:** 2019-01-04

**Authors:** Cole G. Easson, Jose V. Lopez

**Affiliations:** ^1^Department of Biology, Middle Tennessee State University, Murfreesboro, TN, United States; ^2^Halmos College of Natural Sciences and Oceanography, Nova Southeastern University, Dania Beach, FL, United States

**Keywords:** Gulf of Mexico, microbial plankton, 16S rRNA, pelagic, DEEPEND

## Abstract

The Gulf of Mexico (GoM) is a dynamic marine ecosystem influenced by multiple natural and anthropogenic processes and inputs, such as the intrusion of warm oligotrophic water via the Loop Current, freshwater and nutrient input by the Mississippi River, and hydrocarbon inputs via natural seeps and industrial spills. Microbial plankton communities are important to pelagic food webs including in the GoM but understanding the drivers of the natural dynamics of these passively distributed microorganisms can be challenging in such a large and heterogeneous system. As part of the DEEPEND consortium, we applied high throughput 16S rRNA sequencing to investigate the spatial and temporal dynamics of pelagic microbial plankton related to several environmental conditions during two offshore cruises in 2015. Our results show dramatic community shifts across depths, especially between photic and aphotic zones. Though we only have two time points within a single year, observed temporal shifts in microbial plankton communities were restricted to the seasonally influenced epipelagic zone (0–200 m), and appear mainly driven by changes in temperature. Environmental selection in microbial plankton communities was depth-specific, with variables such as turbidity, salinity, and abundance of photosynthetic taxa strongly correlating with community structure in the epipelagic zone, while variables such as oxygen and specific nutrient concentrations were correlated with community structure at deeper depths.

## Introduction

Understanding the ecology, taxonomy and distribution of diverse marine microorganisms (bacteria, fungi, protozoans, and viruses) remains a challenging yet important task. The vastness of the oceans creates highly variable environmental conditions (e.g., aphotic, high/low convection) and diverse microbial niches in three-dimensional space (e.g., marine snow to deep sea hydrothermal vents). Marine microbes play important roles in global biogeochemical cycles (nitrogen fixation, photosynthesis) including the marine microbial loop (Azaml et al., 1983; Giovannoni et al., 1996; Fuhrman, 2009). Due to their intimate connection with the surrounding environment, small changes in abiotic factors such as water temperature, salinity, irradiance, oxygen and oceanic currents can influence microbial community structure and membership (Whitman et al., 1998; King et al., 2013; Sunagawa et al., 2015; Mason et al., 2016; Djurhuus et al., 2017), which may have cascading effects into higher trophic levels. These forces act in varying and complex combinations to structure microbial community composition and function, and thereby driving the dynamics of crucial members of this vast and important ecosystem in the world ocean.

The Gulf of Mexico (GoM) is a dynamic and complex environment whose formation and geologic history helps shape its present-day biology. The GoM basin is relatively deep (>3000 m) with shallow sills that connect the GoM to the Caribbean Sea (Yucatan sill: ∼2000 m) and Atlantic Ocean (Florida sill: ∼700 m), and rich hydrocarbon resources (Wilhelm and Ewing, 1972; Salvador, 1991). Modern-day hydrodynamics play a big role in the uniqueness of the GoM ecosystem. Variable mesoscale features powered by the Loop Current dominate upper level (0–1200 m) seawater dynamics in the GoM (Sturges et al., 1993; Welsh and Inoue, 2000; Hamilton and Lugo-Fernandez, 2001). This feature can lead to the formation of persistent (weeks to months), anti-cyclonic, down-welling eddies composed of warm oligotrophic Caribbean water, leads to a high-degree of vertical mixing causing a well-oxygenated oxygen minimum zone, and may be an important driver of plankton and nekton dispersal as well as providing distinct pelagic habitat (Olson, 1991; Rabalais and Turner, 2001; Rivas et al., 2005; Paulmier and Ruiz-Pino, 2008; Sturges and Kenyon, 2008; Chang et al., 2011; Lindo-Atichati et al., 2012; Wells et al., 2017). Additionally, riverine input from the Mississippi River introduces massive amounts of fresh water laden with agricultural nutrient runoff and terrigenous sediment from the central United States (Rabalais and Turner, 2001; Mason et al., 2016).

The GoM represents one of the most biologically diverse ecoregions (plankton and nekton) among world oceans (Joye et al., 2016; Sutton et al., 2017). The confluence of tropical and temperate climate, as well as mesoscale and terrestrial features, likely contribute to the unique, speciose (nekton) nature of the GoM (Bangma and Haedrich, 2008; Fisher et al., 2016; Sutton et al., 2017). With the GoM’s complex and economically important ecosystem (Adams et al., 2004), understanding its dynamics is crucial, especially in the context of impacts such as massive freshwater and seasonal nutrient inputs by the Mississippi River (Rabalais and Turner, 2001; Gillies et al., 2015), or the massive *Deepwater Horizon* oil spill (DWHOS) and subsequent unprecedented application of dispersant during cleanup (Joye et al., 2016). DWHOS caused massive shifts in microbial plankton leading to a shift in the community to one dominated by taxa that metabolize a variety of hydrocarbons (Redmond and Valentine, 2012; Rivers et al., 2013; Joye et al., 2014). However, the input of large volumes of high nutrient, sediment laden freshwater by the Mississippi River is a more frequent perturbation, which can lead to dramatic shifts in microbial plankton communities and to conditions such as hypoxia that have dramatic effects on coastal marine habitats (Rabalais et al., 1998, 2007; Rabalais and Turner, 2001; Gillies et al., 2015). Due to rapid transport by mesoscale features (e.g., the Loop Current), this plume can extend hundreds of kilometers offshore into the oceanic pelagic environment (Ortner et al., 1995), which may extend the negative effects of this perturbation (e.g., habitat compression) into the offshore pelagic environment (Diaz and Rosenberg, 2008).

At present, we seek to understand the physical and biological dynamics of the GoM ecosystem in the context of microbial plankton communities. Although recent studies have enriched the knowledge of microbial plankton profiles in the GoM (e.g., King et al., 2013; Joye et al., 2014; Mason et al., 2016), there is still much to be learned about this unique ecosystem. Stemming from the DWHOS, the DEEPEND consortium, has initiated more extensive sampling of the GoM oceanic-pelagic environment to include capturing of epipelagic, mesopelagic and bathypelagic communities. This broad scope moves beyond single or a few localized sites (e.g., DWH well site) and has a dynamic component with collections targeting oceanic features such as the Loop Current, Mississippi River plume (Mason et al., 2016), intermediate gradients between the Loop Current and Mississippi River, and assessing temporal variation. This research was conducted as part of the only consortium focused exclusively on the oceanic pelagic ecosystem, from surface to bathypelagic depths. By applying high throughput 16S rRNA gene sequencing technologies and modern analytical methods we provide a profile of the “natural variability” envelope in microbial plankton communities of the offshore pelagic ecosystem.

## Materials and Methods

**FIGURE 1:**
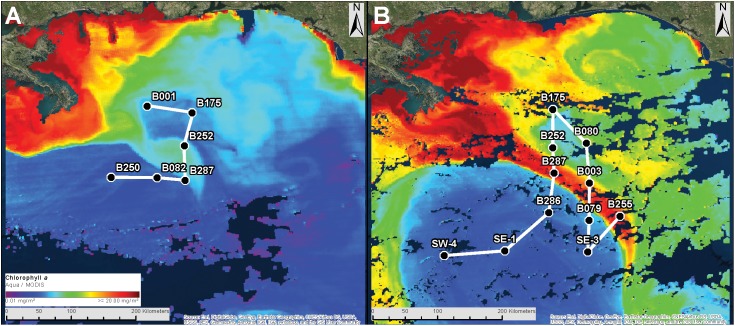
Cruise tracks for DP01 **(A)** and DP02 **(B)**. White lines represent the cruise tracks. Black dots represent the sampling stations for each cruise. The background image represents chlorophyll a concentration measured by MODIS aqua and downloaded using NASA’s Worldview platform (worldview.earthdata.nasa.gov). Red shades peak around 20 mg/m^3^. Imagery is representative of conditions at the beginning of each cruise.

Seawater samples were collected in Niskin bottles deployed on a CTD during two offshore cruises in 2015 in the GoM aboard the *RV Point Sur*. The first 2015 cruise (DP01) occurred May 1–8 and sampled a total of 5 stations, and the second cruise (DP02) occurred August 8–22 and sampled 11 stations (Figure 1 and Supplementary Table S1). These stations are located at every 0.5-degree latitude and longitude in the northern GoM. At each station we collected microbial plankton communities from a maximum of four discrete depths: Ocean surface (SRF; 0–15 m), deep Chlorophyll *a* maximum (DCM; 40–110 m), Oxygen minimum zone (MESO; 450–750 m), and at approximately 1500 m (BATHY), representing three distinct pelagic depth zones (Epipelagic, Mesopelagic, and Bathypelagic). Sampling in the current study is summarized in Supplementary Table S1. During seawater collection, environmental data were simultaneously collected with instruments onboard the CTD. With these instruments, we measured depth (m), temperature (°C), chlorophyll *a* fluorescence (mg/m^3^), salinity (ppt), turbidity (beam c p), chromophoric dissolved organic matter (CDOM; 400 nm absorption/m), seawater density (σ_T_), and O_2_ concentration (ml/L).

One liter of collected seawater was filtered through sterile 0.45 μm filter membranes (Daigger) under low pressure, immediately after CTD retrieval (*n* = 3/station/depth). After filtration, filter membranes were frozen until laboratory analysis at Nova Southeastern University (NSU). At NSU, DNA was extracted from half of each filter membrane, and the remaining half was archived at -80°C. Laboratory preparation and paired-end sequencing of samples was conducted following published protocols from the Earth Microbiome Project (EMP; Gilbert et al., 2014) and using the V4 primers 515F and 806R (Supplementary Methods; Caporaso et al., 2010). At a subset of depths and stations, the filtrate was preserved at 4°C in clean amber bottles for nutrient analysis by the Southeast Environmental Research Center (Miami FL): [Nitrate, (NO_3_^-^) Nitrite (NO_2_^-2^), Ammonium (NH_4_^+^), Total dissolved Nitrogen (TDN), Total dissolved Phosphorus (TDP), Soluble Reactive Phosphorus (SRP), and Dissolved Organic Carbon (DOC); Supplementary Table S1].

Initial bioinformatics processing was accomplished in QIIME (Caporaso et al., 2010). Forward and reverse sequences for all samples were paired and quality filtered (minimum read length fraction ≥ 0.75, maximum bad run length ≤ 3, maximum number of N characters = 0, quality score > 29), followed by operational taxonomic unit (OTU) picking using the default settings in the ‘pick_open_reference_otus.py’ script (see qiime.org/scripts for information on default settings). Taxonomy was assigned to OTUs using the GreenGenes database (DeSantis and Hugenholtz, 2006; Caporaso et al., 2010), and the SILVA database was used as a secondary reference for OTU sequences for instances when GreenGenes provided only limited taxonomic resolution (Pruesse et al., 2012; Quast et al., 2013; Yilmaz et al., 2014).

All statistical analysis was done in (R Core Team, 2016). Analysis was conducted on two datasets: DP02 samples and a temporal dataset from both cruises. Before analysis, singleton and doubleton reads were removed along with OTUs found in fewer than 5% of samples (*n* > 1 samples in DP01; *n* > 6 samples in DP02). These pre-processing steps were done to remove rare features and reduce noise in the data analysis. OTU abundance was transformed to relative abundance (proportional abundance of OTU in whole community) before proceeding with analysis. Our initial analysis investigated differences in community diversity (Inverse Simpson’s index), which assesses community richness and evenness, phylogenetic diversity (Faith’s PD), which measures the total branch length spanned by an individual community (Faith, 1992), and beta-diversity (Bray–Curtis dissimilarity), which analyzes differences in community composition associated with the factors station, depth, and time (cruise) using the R packages vegan and picante (Kembel et al., 2010; Oksanen et al., 2017). Temporal variation was only assessed at three stations (B175, B252, and B287) that were sampled during both cruises in May (DP01) and August (DP02) 2015 to control for potential spatial heterogeneity, but within-cruise variation was assessed at all sampled stations in DP02. Diversity results were checked for normal distribution and heteroskedasticity before proceeding with parametric statistics. An analysis of variance (ANOVA) was used to test for significant differences in diversity and a Permuted multivariate ANOVA (PERMANOVA) was used to assess the significance and effect size of station, depth, and time on microbial community composition. A general linear model (GLM) was used to test for the significant effects of depth as a continuous factor on microbial community diversity (Kembel et al., 2010; Simpson et al., 2016; Oksanen et al., 2017).

Analysis of environmental drivers of microbial community composition was conducted using Canonical Correspondence analysis (CCA) (Oksanen et al., 2017), which has previously been used to investigate correlations among environmental variables and Archaeal communities in the GoM (Tolar et al., 2013). The tested environmental variables included depth, temperature, salinity, chlorophyll *a* fluorescence (Chla), turbidity, CDOM, oxygen, density, and nutrient data where available. The *ordiR2step* forward selection model building function in vegan (see Blanchet et al., 2008; Oksanen et al., 2017) was used to determine the best combination of environmental variables to explain microbial community composition. The output of this function includes a list of selected variables plus the individual and combined effect sizes (corrected explained variance) of each variable. After each model was constructed, a variance inflation test was performed using the *vif.cca* function in vegan. If variance inflation was greater than 10 for any factor, redundant constraints were removed and the model building function was rerun. If two variables were redundant by the *vif.cca* function, the variable with the lower overall explained variance was removed. Due to this process, all CCA models did not include all environmental variables. The significance of model selected CCA axes was tested using a permuted ANOVA (1000 permutations), and the optimal set of environmental variables were determined as the environmental factors that were both selected by the model builder and displayed marginal axis significance. Turbidity data was not collected for DP01, and due to an instrument malfunction, Chla data was not collected for CTD09 in DP02. Therefore, CTD09 was excluded from analysis that included Chla effects.

## Results

A total of 164 samples were collected at 14 stations (SEAMAP stations) during two cruises in May (1–8; DP01) and August (8–21; DP02) of 2015 (Figure 1). DP01 in May 2015 was an abbreviated cruise (8 days) and thus was only analyzed in the context of temporal variation for stations sampled in both cruises. From the raw set of 164 samples (29 from DP01and 135 from DP02), 156 met quality control standards yielding 47,801 unique operational taxonomic units (OTUs). Taxonomic classification using the GreenGenes and SILVA databases revealed 52 phyla and candidate phyla of Bacteria, Archaea, and photosynthetic unicellular eukaryotes (detected via 16S rRNA sequences from chloroplasts).

### Microbial Plankton Dynamics in DP02

The pelagic environment in cruise DP02 (August 2015) was characterized by a strong Loop Current that protruded northward into the northern GoM during the cruise, and a high outflow of the MS River (Figure 1 and Supplementary Figure S1). These two features collided during the DP02 cruise resulting in the transport of low salinity river water into the oceanic pelagic environment (Supplementary Table S1 and Supplementary Figure S1). The cruise track for DP02 was designed to sample across these features and capture a diverse set of environmental conditions. Thus, the dynamics of microbial plankton communities are evaluated in the context of these features, and the potentially unique environments that they represent.

#### Community Diversity

Community diversity (OTU diversity) was strongly influenced by pelagic depth zone, collection station, and the interaction of these two factors (Table 1). Microbial plankton communities on the surface had lower OTU diversity compared to other depths. Phylogenetic diversity (PD) did not show a statistically significant effect of pelagic depths zone. However, it did exhibit a significant effect of collection station, and the interaction of the two factors (Table 1). While pelagic depth zone significantly affected microbial community diversity as a categorical factor, absolute collection depth was only weakly correlated with microbial community diversity (1/*D*; linear regression; df = 1, 131, *F* = 3.89, *R*^2^ = 0.02, *P* = 0.05) and not significantly related with phylogenetic diversity (PD; linear regression; df = 1,131, *F* = 1.44, *R*^2^ = 0.01, *P* = 0.23; Supplementary Figure S2).

**Table 1:** ANOVA and PERMANOVA results from DP02 microbial dynamics statistical tests with independent variables shown.

Dataset/factors	Statistical test	*F*	df	*R*^2^	*p*
**DP02 microbial plankton dynamics**					
*Community diversity*	2-way ANOVA				
Pelagic depth zone		8.25	3		< 0.001
Station		5.39	10		< 0.001
Pelagic zone × station		2.85	19		< 0.001
*Phylogenetic diversity*	2-way ANOVA				
Pelagic depth zone		1.62	3		0.19
Station		3.37	10		< 0.001
Pelagic zone × station		2.87	19		< 0.001
*Community composition*	PERMANOVA				
Pelagic depth zone		101.66	3	0.55	0.001
Station		5.54	10	0.1	0.001
Pelagic zone × station		4.7	19	0.16	0.001
*Phylogenetic composition*	PERMANOVA				
Pelagic depth zone		459.43	3	0.85	0.001
Station		8.71	10	0.05	0.001
Pelagic zone × station		2.84	19	0.03	0.002


*Variables included pelagic depth zone (Surface, Epipelagic, Mesopelagic, and Bathypelagic) and station (collection station in DP02).*

#### Community Composition

Across all samples in DP02, microbial plankton taxonomic community composition (i.e., OTU beta diversity), exhibited substantial variation across pelagic depth zones, stations, and the interaction of these two factors (Table 1). We observed a similar result when microbiome phylogenetics was also considered (i.e., Unifrac dissimilarity) with significant differences among stations, pelagic depth zones, and the interaction of the two factors (Table 1). The phylogenetic dissimilarity results indicate that pelagic depth zone is strongly related to community composition showing that individual depth zones are likely composed of clusters of closely related taxa based on the mean nearest taxon difference (MNTD). Taxonomic and phylogenetic dissimilarity results both indicated distinct communities within each depth zone (pairwise PERMANOVA; *P* = 0.001). Clear stratification by depth zone was evident and accounted for most of the variation among samples.

**FIGURE 2:**
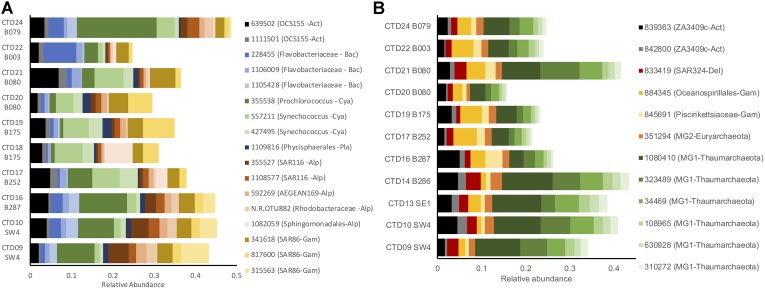
Relative abundance (proportion of OTU sequences) of dominant taxa (>1% mean relative abundance) in the SRF **(A)** and MESO **(B)**. Taxa are grouped by taxonomy: Act, Actinobacteria; Alp, Alphaproteobacteria; Arc, Archaea; Bac, Bacteroidetes; Cya, Cyanobacteria; Del, Deltaproteobacteria; Euk, microbial eukaryotes; Gam, Gammaproteobacteria; Pla, Planctomycetes.

Across these depth zones, microbial plankton communities exhibited major shifts in composition. SRF communities contained a high proportion (relative abundance) of taxa from groups such as (mean proportion ± standard error) *Prochlorococcus* (1 OTU; 0.06 ± 0.02), *Synechococcus* (2 OTUs; 0.06 ± 0.01), SAR86 (Gammaproteobacteria; 3 OTUs; 0.08 ± 0.01), Alphaproteobacteria (5 OTUs; 0.07 ± 0.004), and OCS155 (Actinobacteria; 2 OTUs; 0.05 ± 0.003), Flavobacteriaceae (3 OTUs; 0.05 ± 0.004), and Planctomycetes (1 OTU; 0.01 ± 0.002; Figure 2A and Supplementary Figure S3A). DCM samples were composed of similar taxa in *Prochlorococcus* (2 OTUs; 0.09 ± 0.01), *Synechococcus* (1 OTU; 0.05 ± 0.01), Gammaproteobacteria (2 OTUs; 0.04 ± 0.01), OCS155 (Actinobacteria; 1 OTU; 0.03 ± 0.003), Flavobacteriaceae (1 OTU; 0.02 ± 0.003), Cenarchaeaceae (1 OTU; 0.01 ± 0.002), and Acidimicrobiales (1 OTU; 0.01 ± 0.002) groups, along with eukaryotic phytoplankton (detected via chloroplast sequences) in the groups Haptophyceae (1 OTU; 0.01 ± 0.002) and Stramenopiles (Diatoms: 1 OTU; 0.01 ± 0.002; Supplementary Figures S3A, S4A). MESO communities collected at the oxygen minimum zone were highly divergent from shallower samples and were dominated by Marine Group 1 Thaumarchaeota (6 OTUs; 0.18 ± 0.004), Marine Group 2 Euryarchaeota (1 OTU; 0.01 ± 0.001), Gammaproteobacteria (2 OTUs; 0.05 ± 0.003), ZA3409c-Actinobacteria (2 OTUs; 0.04 ± 0.003), and SAR324-Deltaproteobacteria (1 OTU; 0.02 ± 0.002; Figure 2B and Supplementary Figure S3B). BATHY communities were distinct from all other depths and contained high percentages of Gammaproteobacteria (4 OTUs; 0.08 ± 0.004), Marine Group 1 Thaumarchaeota (2 OTUs; 0.05 ± 0.004), Alphaproteobacteria (3 OTUs; 0.04 ± 0.004), SAR324-Deltaproteobacteria (1 OTU; 0.03 ± 0.004), ZA3409c-Actinobacteria (1 OTU; 0.02 ± 0.003), Rhodothermaceae (1 OTU; 0.01 ± 0.01), Marine Group 2 Euryarchaeota (1 OTU; 0.01 ± 0.001), and Flavobacteriales (1 OTU; 0.01 ± 0.01; Supplementary Figures S3C, S4B).

Microbial plankton community similarity showed a weak relationship with spatial distance (latitude and longitude; mantel test: *r* = 0.12, *P* = 0.001) that was not significant when microbiome phylogenetics were considered (mantel test: *r* = 0.01, *P* = 0.55). Similar results were observed when depth effects were first removed for both taxonomic and phylogenetic dissimilarity (partial Mantel test; taxonomic dissimilarity: *r* = 0.18, *P* = 0.001; phylogenetic dissimilarity: *r* = 0.02, *P* = 0.30). Overall, community composition results indicated substantial shifts in community composition across collection depths, while spatial (among site) differences tended to be much weaker. These results combined with the low variance explained by collection site (PERMANOVA results) suggest that simple microbial plankton dispersal is not a primary factor in community composition, as spatially similar samples within the same depth zone can have widely disparate compositions. For example, microbial plankton on the seawater surface (SRF) at station B287 were more similar to SRF samples at station SW-4 (∼180 km away) than to those collected at stations B252 and B003 (∼50 km away; Figure 1 and Supplementary Figure S1).

Several OTUs contributed to pairwise differences between depth zones (SIMPER analysis, 999 permutations; Supplementary File S1). Supplementary Figure S3 highlights 17 taxa that dominated samples in different depth bins and explained a large portion of the compositional differences. Major phylum-level variation among depths included shifts from photoautotrophic taxa in the SRF and DCM (e.g., Cyanobacteria, Eukaryotic phytoplankton, SAR116 Gammaproteobacteria) to chemoautotrophic taxa in the MESO and BATHY (e.g., Marine Group I and II Thaumarchaeota and Euryarchaeota, Deltaproteobacteria, Gammaproteobacteria; Figure 2 and Supplementary Figure S5).

#### Environmental Effects on Community Composition

Canonical correspondence analysis (CCA) was used to investigate environmental drivers of microbial plankton dynamics, similar to previous GoM microbial plankton studies (Tolar et al., 2013). CTD profiles for Temperature-Depth, Density-Depth, Salinity-Depth, and Temperature-Salinity for DP02 are located in Supplementary Figure S6.

**FIGURE 3:**
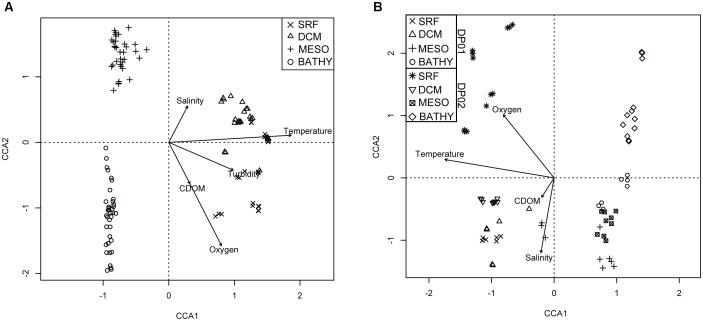
Triplots of top constrained axes for CCA analysis of DP02 **(A)** and temporal comparison of shared DP01-DP02 stations **(B)**. Symbols represent unique depth zones in each cruise. Vectors in plots represent direction and magnitude of influence for labeled environmental parameter.

All CCA results are summarized in Table 2. The CCA model that included all samples from DP02 explained approximately 23% of the sample variance, and temperature was the most important environmental variable, explaining more than triple the variance of the next term in the model (Figure 3A). Much of the overall sample variance is not explained by the selected variables, suggesting that either unmeasured parameters may be driving microbial community variability and/or the effect of individual environmental parameters was variable across depths. To test the latter, scenario, we split the samples by depth zone, which was previously shown as an important factor for community composition.

**Table 2:** Canonical correspondence analysis (CCA) results from DP02.

CCA results	Sig. variables	Exp. variance
**DP02 microbial plankton dynamics**		
**All samples**	**All variables**	**0.23**
	Temperature	0.14
	Oxygen	0.04
	Turbidity	0.03
	Salinity	0.02
		
**Surface (SRF)**	**All variables**	**0.35**
	Turbidity	0.2
	Temperature	0.06
	Oxygen	0.06
	Salinity	0.03
**Epipelagic (DCM)**	**All variables**	**0.4**
	Chlorophyll *a*	0.16
	CDOM	0.13
	Salinity	0.07
	Temperature	0.04
**Mesopelagic (MESO)**	**All variables**	**0.11**
	Depth	0.04
	Turbidity	0.04
	CDOM	0.02
	Oxygen	0.02
**MESO w/Nutrient**	**All variables**	**0.34**
	Oxygen	0.13
	Depth	0.08
	Ammonium	0.05
	Salinity	0.08
**Bathypelagic (BATHY)**	**All variables**	**0.29**
	SRP	0.08
	Nitrite	0.07
	Depth	0.05
	Salinity	0.04
	Oxygen	0.03
	CDOM	0.02
	DOC	0.01


*Only significantly correlated environmental variables (Sig. variables) are shown along with the effect size (Exp. Variance) of each individual variable and the total effect size (all variables). Environmental variables included: depth, temperature, salinity, chlorophyll a fluorescence, turbidity, CDOM, oxygen, density, nitrate, nitrite, ammonium, soluble reactive phosphorus (SRP).*

**FIGURE 4:**
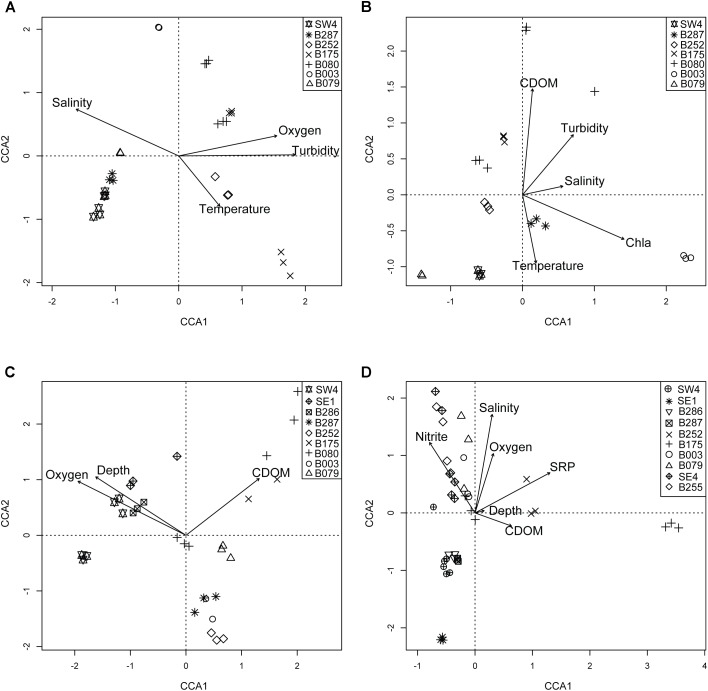
Triplots of individual pelagic depth zones in DP02: SRF **(A)**, DCM **(B)**, MESO **(C)**, BATHY **(D)**. Symbols represent unique sampling stations. Vectors in plots represent direction and magnitude of influence for labeled environmental parameter. Specific environmental influences varied across depths, and thus different combinations of environmental variables are shown for each depth bin.

**FIGURE 5:**
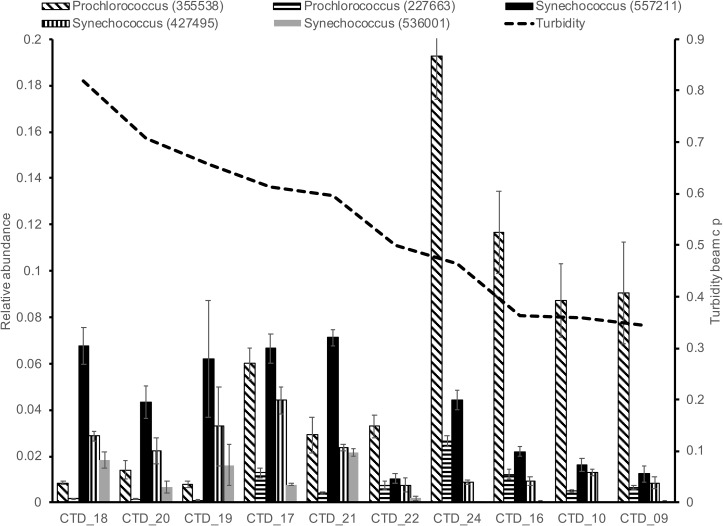
Relative abundance of five Cyanobacteria taxa in relation to a gradient of turbidity. Higher turbidity in DP02 was indicative of influence of the Mississippi River in the pelagic environment.

For surface (SRF) microbial plankton communities, the CCA model variables accounted for approximately 35% of the overall sample variance. Turbidity was the most important variable, and this variable alone accounted for more than half of all variance explained by environmental and spatial variables (Figure 4A). Across sampling stations, the highest turbidity was observed at B175 and B080 while the lowest turbidity was observed at SW4 (Figures 1B, 5 and Supplementary Figure S1). CDOM and Chl*a* were removed from the model due to redundancy and depth was not considered because it could not be determined to meter precision at this depth due to ocean swell.

The epipelagic (DCM) samples, collected at the chlorophyll maximum peak, showed a relatively strong correlation with environmental variables, which explained approximately 40% of the overall sample variance (Table 2 and Figure 4B).

Mesopelagic microbial plankton communities, which were collected at the oxygen minimum zone, were only weakly correlated with environmental and spatial variables (11% of overall sample variance; Table 2). Of the environmental variables, the depth of the oxygen minimum zone was the most important (Figure 4C), and downwelling forces in anticyclonic features such as the Loop Current can push features such as chlorophyll maximums and oxygen minimums deeper in the water column (Supplementary Figure S7). For 18 out of 37 MESO samples, nutrient data were collected (Supplementary Table S1). These samples were from CTD09-CTD17 (SW4 – B252), which represented an onshore gradient and was hypothesized to span a gradient from the Loop Current to residual GoM water (Figure 1 and Supplementary Figure S1). Substantially more variance was explained in these limited samples (34% of sample variance; 34% with Lat-Lon variables) compared to the larger MESO sample dataset. Oxygen concentration was the most important variable, and notably, the nutrient, ammonium was also observed to be a significant environmental variable (Supplementary Figure S8 and Table 2).

Analysis of BATHY samples showed that the measured environmental variables, which included nutrient data for all stations, explained approximately 29% of the overall sample variance. Soluble reactive phosphorus (SRP) and nitrite concentration were the most important individual variables. The most divergent samples were from CTD18 at station B175, which occurs within the DeSoto Canyon benthic feature (Figure 4D).

### Temporal Change: DP01 – DP02 at Stations B175, B252, and B287

Microbial plankton diversity varied significantly among collection stations, pelagic depth zones, between cruises, and the interaction of each pair of variables (Table 3). These results were mainly driven by low diversity in the surface (SRF) samples from DP02 (Tukey’s HSD *P* < 0.05). While we observed significant differences in diversity associated with pelagic depth zone, absolute collection depth was not significantly related to microbial community diversity (GLM, df = 1,56, *F* = 0.001, *R*^2^ = -0.02, *P* = 0.98). Phylogenetic diversity also varied significantly among collection station, pelagic depth zones, between cruises, for the interaction of station and pelagic depth zone, and for the interaction of all three factors (Table 3). Phylogenetic diversity did not vary significantly with absolute collection depth (linear regression; df = 1, 56, *F* = 0.26, *R*^2^ = -0.01, *P* = 0.61).

**Table 3:** ANOVA and PERMANOVA results from DP01–DP02 temporal microbial dynamics statistical tests with independent variables shown.

Dataset/factors	Statistical test	*F*	df	*R*^2^	*p*
**DP01–DP02 temporal change**					
*Community diversity*	3-way ANOVA				
Pelagic depth zone		5.3	3		0.004
Station		5.99	2		0.005
Cruise		8.76	1		0.005
Pelagic zone × station		3.16	5		0.02
Station × cruise		4.81	1		0.03
Pelagic zone × cruise		6.37	3		0.001
*Phylogenetic diversity*	3-way ANOVA				
Pelagic depth zone		3.41	3		0.03
Station		13.18	2		< 0.001
Cruise		20.86	1		< 0.001
Pelagic zone × station		4.31	5		0.003
Pelagic zone × station × cruise		5.44	2		0.008
*Community composition*	PERMANOVA				
Pelagic depth zone		38.93	3	0.48	0.001
Station		6.85	2	0.06	0.001
Cruise		14.94	1	0.06	0.001
Pelagic zone × station		3.94	5	0.08	0.001
Station × cruise		4.16	1	0.02	0.004
Pelagic zone × cruise		8.1	3	0.1	0.001
Pelagic zone × station × cruise		4	2	0.03	0.001


*Variables included pelagic depth zone (Surface, Epipelagic, Mesopelagic, and Bathypelagic), station (collection station in DP01 and DP02), and cruise (DP01 or DP02).*

We observed significant effects of cruise, collection station, pelagic depth zone, the interaction of each pair of factors, and the interaction of all three factors on microbial plankton community composition (Table 3). Most of the variance among samples was explained by pelagic depth zone or interactions with this factor (∼69%), while collection site and cruise only accounted for ∼14% of the total sample variance.

#### Environmental Effects

The CCA model with environmental variables explained approximately 25% of the overall sample variance, with temperature representing the most important environmental variable (Table 4 and Figure 3B). Temperature variation among cruises was only apparent for the epipelagic zone, and this variable appears to be most related to changes among cruises for seasonally affected depths.

**Table 4:** Canonical correspondence analysis (CCA) results from DP01–DP02 temporal analysis.

CCA results	Sig. variables	Exp. variance
**DP01–DP02 temporal change**		
	**All variables**	**0.25**
	Temperature	0.13
	Salinity	0.05
	Oxygen	0.04
	CDOM	0.02


*Significantly correlated environmental variables (Sig. variables) are shown along with the individual and total effect sizes (Exp. Variance). Environmental variables included: depth, temperature, salinity, chlorophyll a fluorescence, oxygen, CDOM, and density.*

## Discussion

The pelagic ecosystem challenges in-depth, systematic studies because of its sheer size, wide spatial and temporal dynamics, and influences from complex combinations of oceanic (Lindo-Atichati et al., 2012; Djurhuus et al., 2017), terrestrial (Mason et al., 2016), benthic (Rivers et al., 2013; Joye et al., 2014; Rakowski et al., 2015), biological (Lam and Kuypers, 2011; Lima-Mendez et al., 2015), and climatological forces (Sunagawa et al., 2015). Our study builds on previous microbial plankton biogeography research by combining elements that were often separate to gain a broader view of microbial plankton dynamics in this particular GoM region. We aimed to move beyond the simple characterization of GoM microbial plankton, accomplished in previous research (e.g., King et al., 2013; Mason et al., 2016), and leverage our dataset to better understand microbial plankton dynamics in the oceanic pelagic environment across depth, time, and a host of environmental variables as well as in the context of prominent features in the oceanic pelagic environment during sample collection (Johnston et al., unpublished). Passively distributed microbial plankton communities varied in diversity and composition across depth zones but exhibited complex spatial patterns of variation among collection stations. The specific forces of environmental selection were depth-dependent, and ranged from turbidity, salinity, and season (cruise) at the surface to nutrient concentration and depth in the BATHY samples. These depth-dependent forces may provide clues as to the broader drivers (e.g., Mississippi River and Loop Current) of community dynamics (Mason et al., 2016; Djurhuus et al., 2017).

Microbial biogeography remains a key area of interest in earth and oceanographic studies, with recent expeditions such as TARA Oceans making substantial contributions to our understanding of how environmental forces and dispersal drive biogeographic patterns of microbial plankton worldwide (Armbrust and Palumbi, 2015; Sunagawa et al., 2015). Previous research on pelagic microbial communities emphasized the importance of depth, demonstrating that this complex factor is a powerful driver of microbial plankton composition and function due to changes in several physio-chemical parameters (King et al., 2013; Tolar et al., 2013; Armbrust and Palumbi, 2015). Sunagawa et al. (2015) showed that stratification of microbial plankton communities was a global phenomenon, and observed distinct communities at SRF, DCM, and MESO depths. Similarly, the current study found vertical stratification patterns in microbial plankton composition. Shallow communities (SRF and DCM) were dominated by several photoautotrophic taxa (e.g., *Prochlorococcus, Synechococcus*, SAR86-Gammaproteobacteria, SAR116-Alphaproteobacteria, eukaryotic phytoplankton), while MESO and BATHY communities, while distinct from each other, were dominated by potentially chemoautotrophic taxa such as Marine Group 1 (MG1) Thaumarchaeota, Deltaproteobacteria, and Gammaproteobacteria (Figure 2). These shifts in composition suggest dramatic shifts in community function, which has been explicitly measured in other studies (Tolar et al., 2013; Lima-Mendez et al., 2015), as members of these communities have adapted to a range of niches specific to the environment in these vertical strata. The apparent absence of the common SAR11 taxa in our dataset is likely due to a known primer bias against these taxa that also may underestimate Thaumarchaeota abundance, though these latter taxa still dominated samples from the oxygen minimum zone (Parada et al., 2016). Parada and Fuhrman (2017) went on to characterize the dynamics of Euryarchaea Marine Group II (MGII) from the surface to 890 m depth and found dynamic assemblages of MGI-*Nitrospina* assemblages as part of the 15 years + San Pedro Ocean Time-series (SPOT) off the coast of Los Angeles.

Geographic distance-decay similarity relationships are observed at large (up to 5000 km) (Fuhrman, 2009; Tittensor et al., 2010; Raes et al., 2011; Sunagawa et al., 2015) and small scales (∼1 km; Martiny et al., 2011). At intermediate scales (∼100 km), especially in the pelagic environment, these relationships can be complicated by high dispersal rates and a heterogenous environment driven by ocean currents. In the current study, distance-decay relationships were weak even when depth effects were first considered. Patterns of diversity and composition across stations were complex and combined with the weak relationship to spatial distance, may point to active environmental selection by spatially heterogeneous forces in GoM microbial plankton communities rather than simple dispersal limitation. The different pelagic features sampled in DP02 may present unique environmental selection pressures on microbial plankton communities even when these features interact (Supplementary Figure S1).

Many oceanographic studies, including the current study, provide evidence for environmental structuring of microbial plankton communities at specific depths (Bianchi et al., 2011; Dubinsky et al., 2013; King et al., 2013; Ortmann and Ortell, 2014; Gillies et al., 2015; Sunagawa et al., 2015), sometimes tied to specific habitat features (Hahnke et al., 2013; Mason et al., 2016). In previous research, broad community gradients were often absent, and shifts in community composition were attributed to factors such as riverine inputs (Mason et al., 2016), hydrocarbon inputs (Joye et al., 2014, 2016; Scott et al., 2014; Rakowski et al., 2015), and distinct oceanic water masses (Hahnke et al., 2013; Djurhuus et al., 2017). Discerning specific influences of features can be difficult, especially at feature boundaries where different water masses mix. In the current study, we did not attempt to determine the precise spatial extent of oceanic features, but rather utilize some well-known markers of these features such as low salinity (Mississippi River) and the presence of the subtropical underwater current (Loop Current) to give broader context to our results.

We observed depth-specific combinations of environmental drivers of microbial plankton communities. In the SRF communities in DP02, microbial plankton community composition exhibited spatial heterogeneity largely related to changes in turbidity. Stations where turbidity was higher and salinity was lower (e.g., B175, B080) contained distinctly different communities compared to those with lower turbidity, and oceanic salinity (e.g., SW4, Figures 4A, 5). Taxonomic shifts across these environmental gradients show variation in disparate groups of closely related taxa such as two likely photoautotrophic groups that included five Cyanobacteria (2 *Prochlorococcus* spp. and 3 *Synechococcus* spp.) and six SAR86 Gammaproteobacteria (Figure 5 and Supplementary Figure S7). Interestingly, we did not observe overall decreases in these taxa groups across the turbidity gradient, but rather shifts within closely related taxa groups. One hypothesis is that taxa within these groups may represent different ecotypes that have different preferred light environments, but the current study cannot specifically ascribe these shifts solely to changes in irradiance. Many previous ocean microbial ecology studies have characterized and evaluated shifts in microbial plankton at broad taxonomic levels (e.g., Phylum and Class), and such coarse binning of taxa would not have captured the spatial dynamics in the current study.

Previous studies in the GoM have suggested that the Mississippi River has higher alpha diversity and may act as a seed bank of microbial taxa for the pelagic environment (Mason et al., 2016). In the current study, a clear signal of the Mississippi River is present (i.e., low salinity, high turbidity water), and distinct community composition was associated with these environmental factors. However, we found no evidence to support the “seed bank” hypothesis. Microbial plankton samples that exhibited the lower salinity (CTD17, CTD18, CTD19; Supplementary Table S1), indicative of Mississippi River intrusion (Hu et al., 2005) did not show significantly higher diversity. Moreover, subsequent collections (12 h later) at the same stations where salinity was more reflective of oceanic water (CTD18–CTD19 at B175 and CTD20–CTD21 at B080) showed no shifts in community diversity. The different results in the current study could be partly due to sampling bias, as Mason et al. (2016) only sampled a single station in the pelagic environment, compared to five coastal stations. Alternatively, and perhaps more critically, the seed bank claim does not appear to have statistical support in Mason et al. (2016), and the results of the current study suggest that this hypothesis is unsubstantiated. Based on our results, the Mississippi River may introduce unique taxa to the pelagic environment (Figure 4A, stations B252, B175, and B080), which could be useful as tracers of Mississippi River intrusion, but it does not appear to boost microbial plankton community diversity in the broader oceanic environment.

Sampling of the DCM communities targeted the depth of the maximum subsurface chlorophyll a concentration, which is vertically positioned by a variety of forces including light attenuation, nutrients, and ocean currents (Ortmann and Ortell, 2014). DCM samples were collected within the pycnocline (Supplementary Figure S5), at densities ranging from 23.7 σ_T_ to 25.6 σ_T_, and we observed the main drivers of community composition at this depth to be phytoplankton abundance (Chla), which was highest at stations B287 and B003, CDOM, and temperature. Changes in these environmental parameters were accompanied by shifts in taxa within groups such as Cyanobacteria, Gammaproteobacteria, Flavobacteria, Alphaproteobacteria, Verrucomicrobia, and eukaryotic phytoplankton. A close examination of the trends in these samples reveals that the deepest DCM communities had the lowest CDOM concentrations and were the warmest (SW4, B079, and B287). Warm oligotrophic water is typical of the Loop Current origin water in the GoM, and the down-welling force of this anticyclonic feature could be responsible for the observed trends in environmental parameters as the Loop Current was a prominent feature during the DP02 cruise. The Loop Current can affect the abundance of pelagic nektonic fauna at these depths (Biggs, 1992; Biggs and Müller-Karger, 1994; Lindo-Atichati et al., 2012; Wells et al., 2017), and for microbial plankton, may represent a powerful force for both environmental selection and novel taxa dispersal. However, defining the boundaries of mesoscale features such as the Loop Current is difficult, so teasing apart the direct influences of this feature remain difficult.

MESO communities were sampled at the oxygen minimum zone (OMZ), which in the GoM is generally well oxygenated compared to other world ocean OMZs (Tyson and Pearson, 1991; Lam et al., 2009; Lam and Kuypers, 2011), never reaching dysoxic concentrations (<2.0 ml L^-1^; (Tyson and Pearson, 1991; Lam et al., 2009; Lam and Kuypers, 2011). Thus, many anoxic and suboxic metabolic processes may not occur in GoM microbial plankton communities at this depth, under normal conditions. The dominant taxa in this layer in the current study were Archaea (mostly MG1 Thaumarchaeota; Figures 2, 5), similar to other GoM studies (Tolar et al., 2013; Bristow et al., 2015), and previous research in the GoM showed a high abundance of ammonia oxidation genes associated with MG1 Thaumarchaeota presence (Tolar et al., 2013; Swan et al., 2014). The less dramatic OMZ in the GoM likely favors processes that consume oxygen, since it is not a limiting resource (Lam et al., 2009). This may explain why in the current study, the dominant taxa in the OMZ appear to be ammonia oxidizers, and not taxa that would perform anoxic processes as observed in more severe OMZs in the world oceans (Lam et al., 2009; Lam and Kuypers, 2011; Hewson et al., 2014; Ganesh et al., 2015). Shifts in taxa across sites in this zone were only weakly correlated with measured variables, which generally exhibited lower variability compared to shallow depths. Despite this weak correlation, several Thaumarchaeota taxa were variable across stations, which may support differential responses of MG1 ecotypes to environmental variables (Tolar et al., 2013) that were not measured in the current study.

Oceanographic circulation in the GoM is typically considered a two-level system (Hamilton, 1990, 2009; Sturges et al., 1993; Welsh and Inoue, 2000; Cardona and Bracco, 2016), with the surface to approximately 1200 m (which includes SRF, DCM, and MESO samples) comprising the upper level and bathypelagic zone (BATHY samples) comprising the lower level. This lower level of circulation is semi-isolated and only indirectly linked to the dynamics at shallower depths, though shallower water features such as the Loop Current help drive circulation in the bathypelagic zone (DeHaan and Sturges, 2005; Rivas et al., 2005; Chang et al., 2011; Bracco et al., 2016). Seawater in the bathypelagic zone has an estimated residence time of 250 years, and only periodically exchanges through the Yucatan peninsula back into the Caribbean (Rivas et al., 2005). In the current study, BATHY communities were distinct from all other communities, showing high phylogenetic diversity, and a strong correlation with SRP concentration, which represents crucial nutrient to marine microbial plankton (Karl, 2014). SRP concentration did not show a clear spatial pattern among stations, although the highest SRP concentrations were observed in two samples at station B175, located nearest the northern coast, and within the De Soto Canyon, whose unique structure and flow regime may play a role in re-suspending sediment with elevated SRP (Posamentier, 2003). Environmental selection in microbial plankton at this depth appears complex and likely comprises a combination of circulation patterns, heterogeneous distribution and composition of particles sinking from shallower depths, benthic topography, and hydrocarbon seepage that drive nutrient dynamics (Hewson et al., 2006; Rivers et al., 2013). Further research is needed to tease apart the specific forces that control and affect these deep-water communities.

## Conclusion

Our results add to the growing body of knowledge of GoM microbial plankton and expand our understanding by more extensively sampling the oceanic pelagic environment across broad spatial, temporal and depth ranges (14 stations, 164 samples, depth range 2–1500 m). Environmental selection in microbial plankton communities is evident, especially across variable seawater depths, and evaluation of environmental selection in the current study was best achieved in a depth-dependent context. This depth-dependent context showed complex selection that appears related to broader oceanic features in the GoM including the Loop Current and Mississippi River plume. Additionally, we observed several examples of shifts in closely related taxa across environmental gradients and stations, which may challenge common practices in ocean microbial studies of evaluating microbial plankton communities at coarse taxonomic levels if these closely related taxa shifts are ecologically meaningful. Taken together, our results add valuable insight into the forces that structure oceanic-pelagic microbial plankton communities in the GoM and provide a foundation for future studies investigating topics such as the effects of microbe-microbe networks on the structure microbial plankton communities, and the use of these communities as tracers of converging water masses in the GoM.

## Author Contributions

CE and JL contributed to the conception and the design of the study. CE organized the datasets, performed the statistical analysis, wrote the first draft of the manuscript, and revised the manuscript. All authors contributed to the final manuscript revision, read, and approved the submitted version.

## Conflict of Interest Statement

The authors declare that the research was conducted in the absence of any commercial or financial relationships that could be construed as a potential conflict of interest.
