# Supplementary figures and images for: Depth-Dependent Environmental Drivers of Microbial Plankton Community Structure in the Northern Gulf of Mexico
Source: Front Microbiol. 2019 Jan 4;9:3175. doi: 10.3389/fmicb.2018.03175 (PMC6328475; doi:10.3389/fmicb.2018.03175)

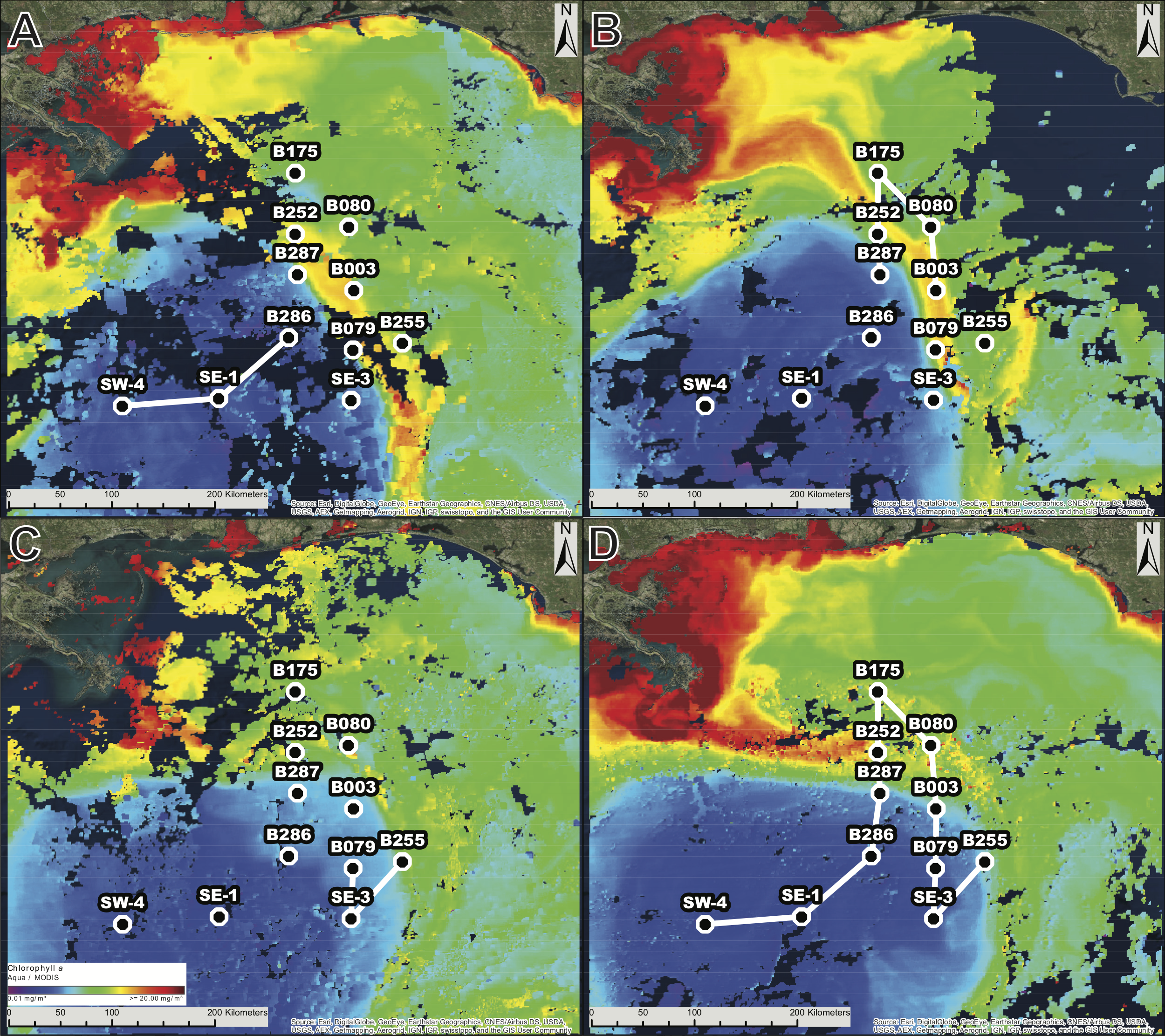

Supplement: Supplementary file 1 [file Image_1.TIFF]

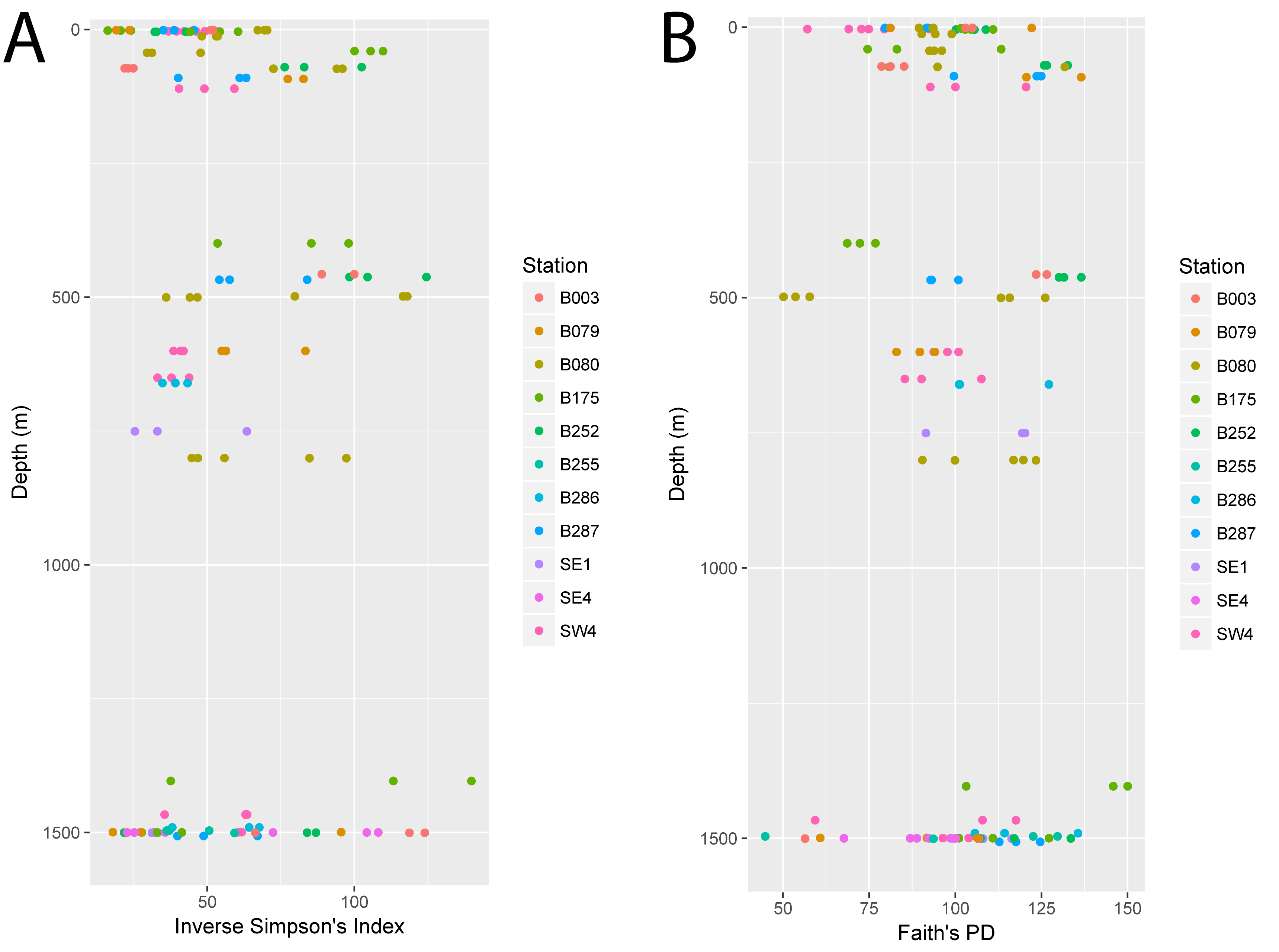

Supplement: Supplementary file 2 [file Image_2.TIF]

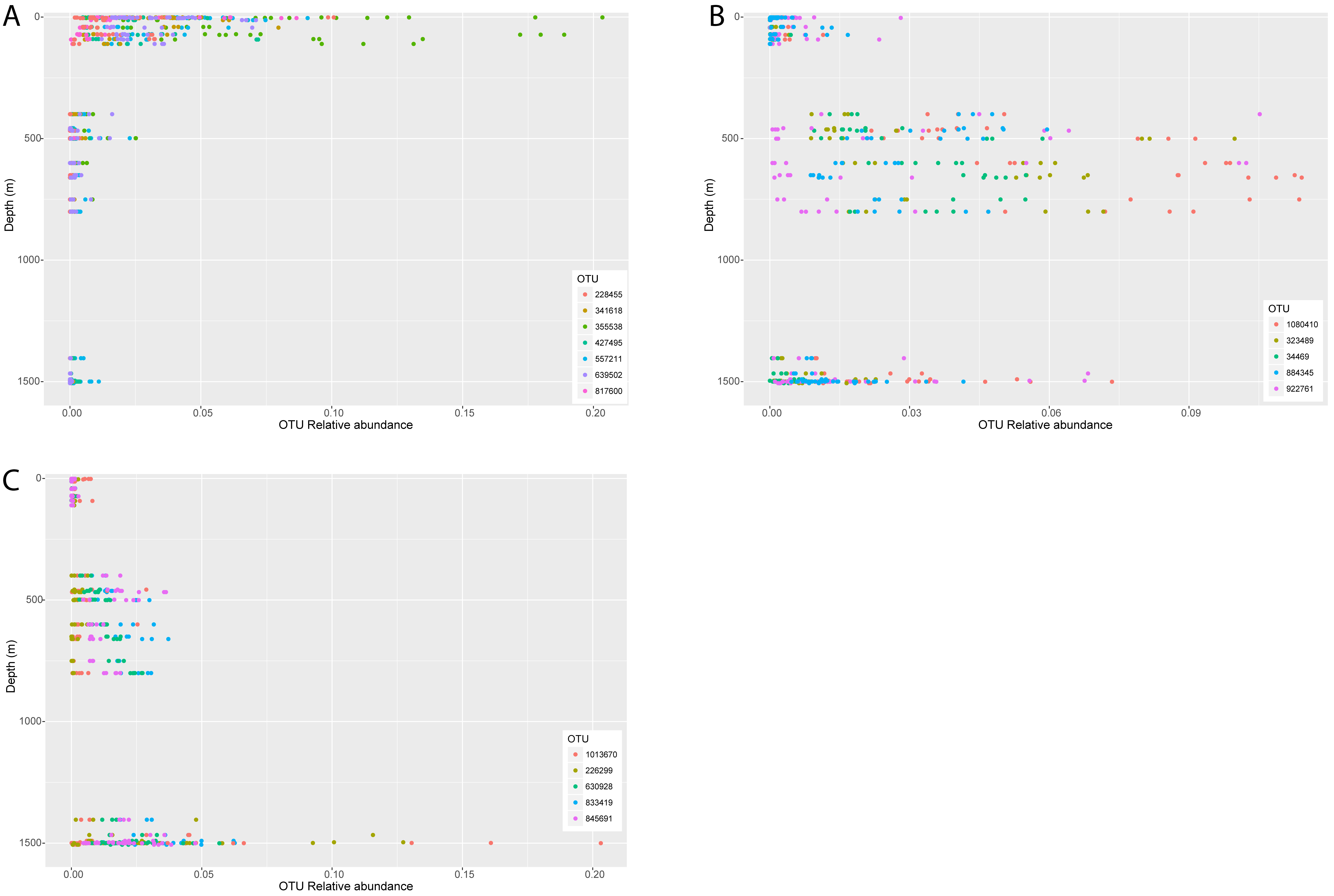

Supplement: Supplementary file 3 [file Image_3.TIF]

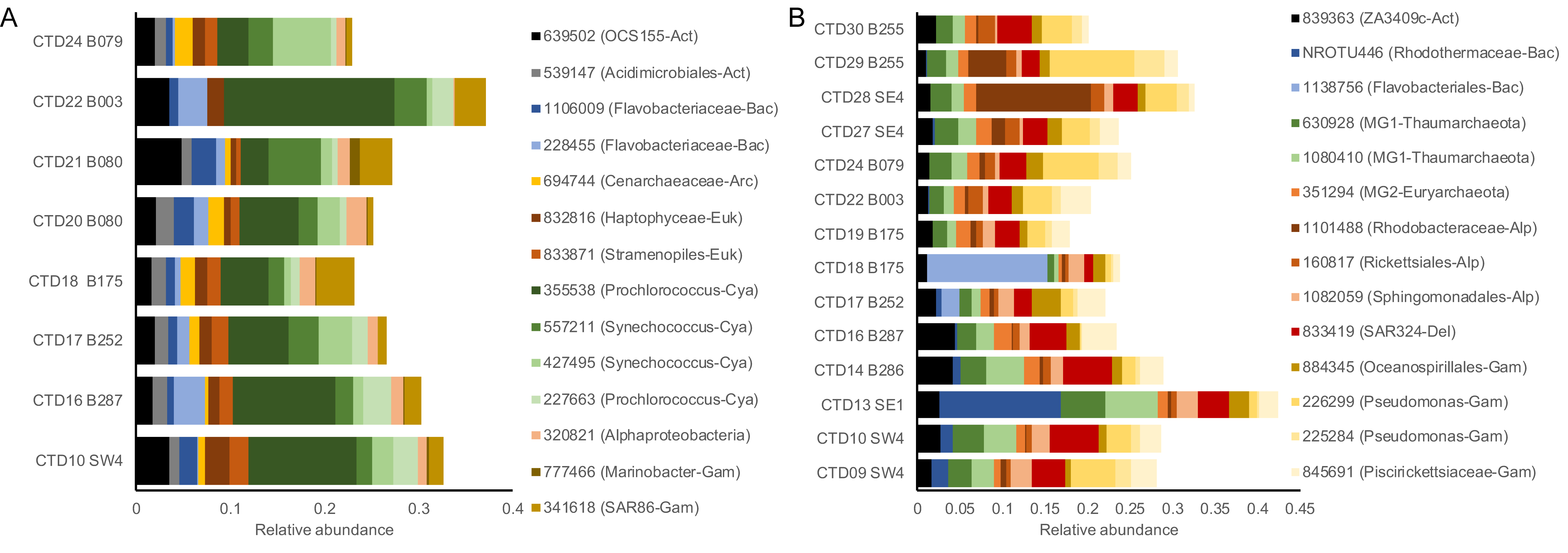

Supplement: Supplementary file 4 [file Image_4.tif]

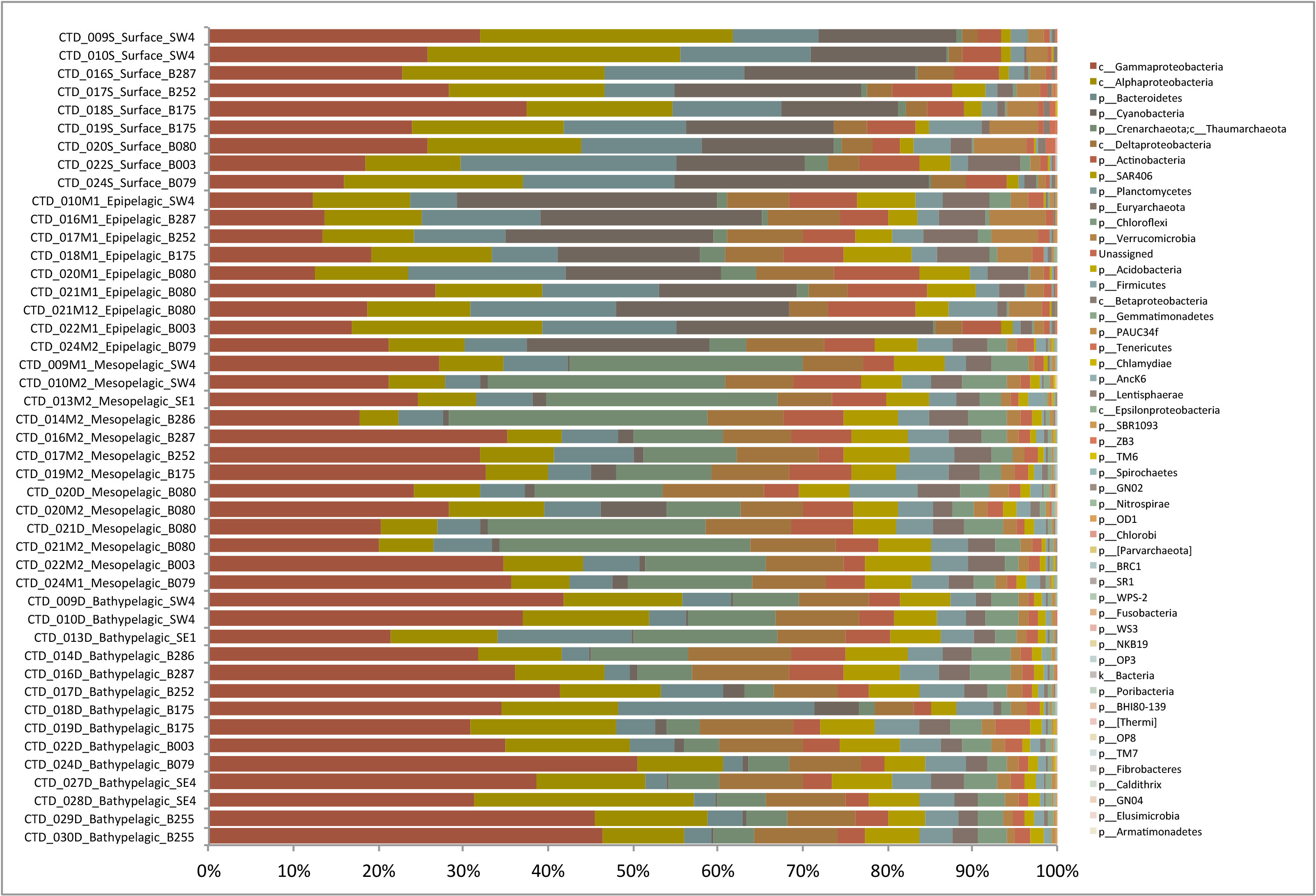

Supplement: Supplementary file 5 [file Image_5.JPEG]

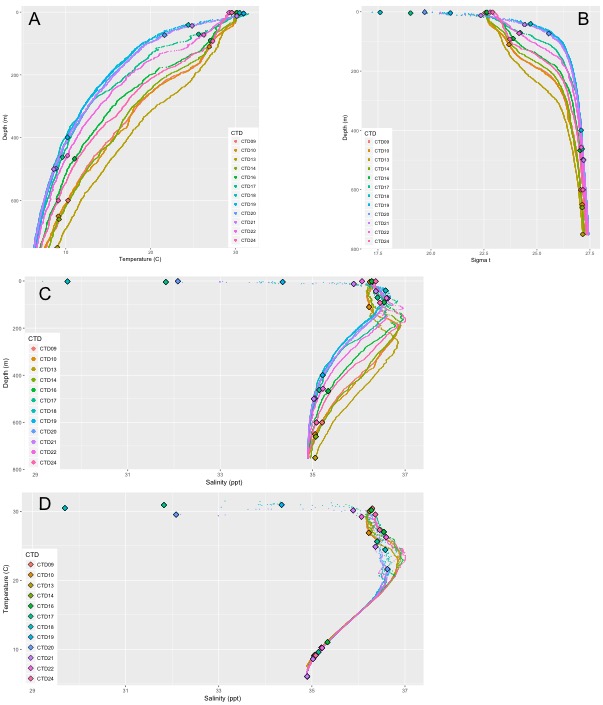

Supplement: Supplementary file 6 [file Image_6.JPEG]

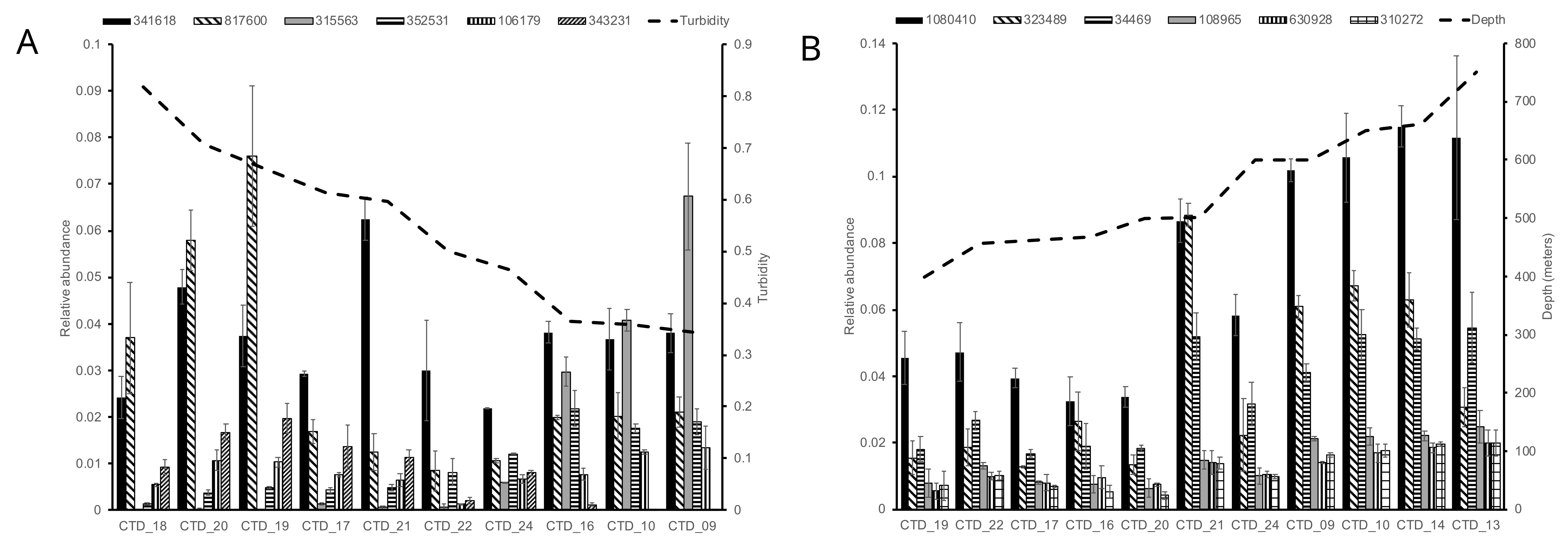

Supplement: Supplementary file 7 [file Image_7.JPEG]

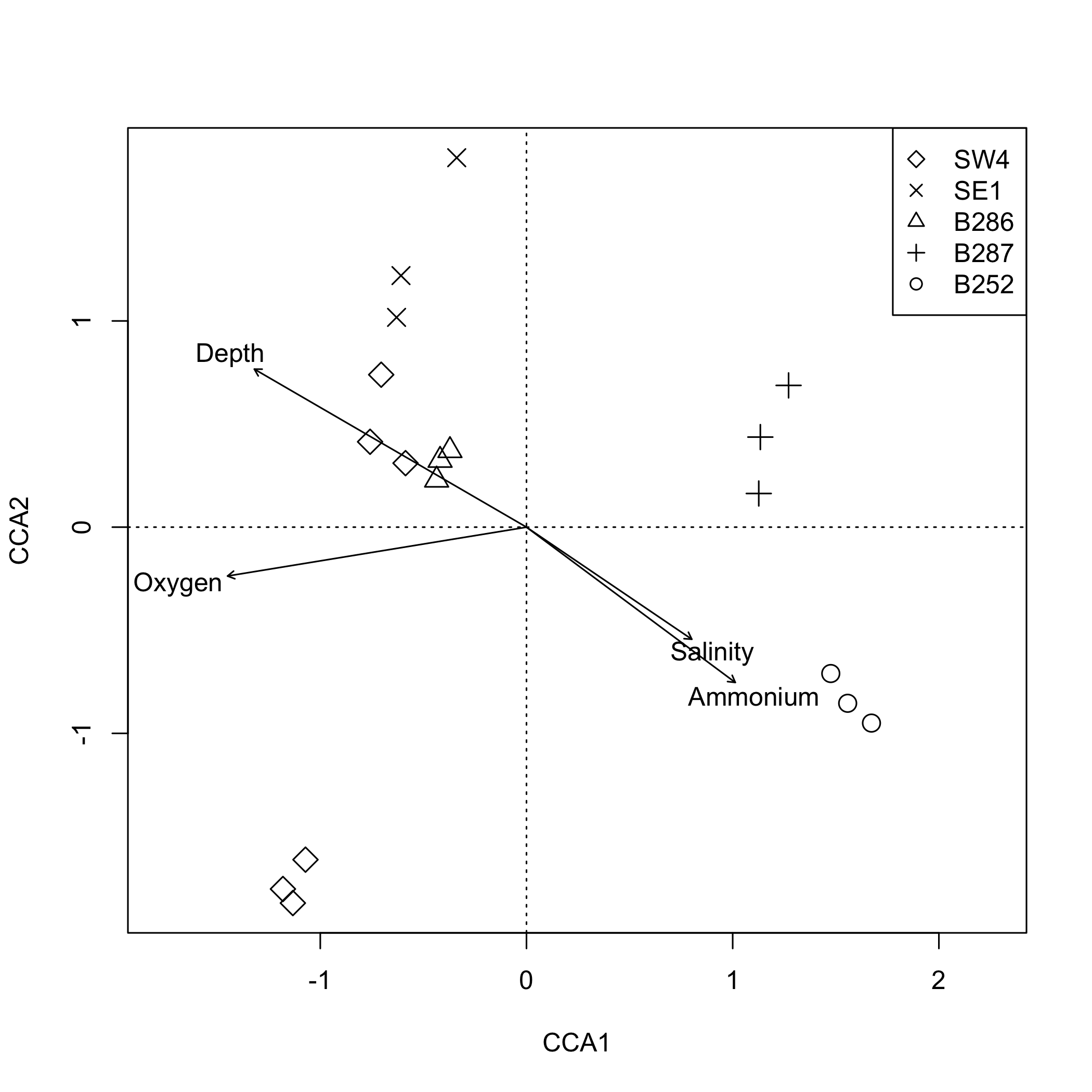

Supplement: Supplementary file 8 [file Image_8.TIFF]
