# Supplementary material for: Depth-Dependent Environmental Drivers of Microbial Plankton Community Structure in the Northern Gulf of Mexico
Source: Front Microbiol. 2019 Jan 4;9:3175. doi: 10.3389/fmicb.2018.03175 (PMC6328475; doi:10.3389/fmicb.2018.03175)
Supplement: Supplementary file 9 [file Data_Sheet_1.PDF]

Supplemental methods

Laboratory preparation and sequencing of collected seawater samples.

Total genomic DNA was extracted from the half filter membrane using the MoBio Powerlyzer DNA extraction kit and following the manufacturers protocol. After DNA extraction, we performed Polymerase Chain Reaction (PCR) following the protocols published by the Earth Microbiome Project (EMP). Briefly, the 16S rRNA V4 region was amplified using the primers 515F and 806R** (Caporaso et al., 2010, 2012), the 2.5x 5Prime HotMasterMix PCR solution, sample DNA, and PCR water. Each primer included Illumina adapters, and each reverse primer (806R) contained a unique 12 base pair barcode. Each sample was assigned a unique barcode to allow for multiplexing and later bioinformatics separation of individual samples. The amplification protocol on the thermal cycler was 94° C (3 min), 35 cycles of 94° C (45 sec)- 50° C (60 sec)- 72° C (90 sec), and 72° C (10 min). Samples were checked on agar gel with a 100 base pair ladder (Promega) to confirm amplification success and correct amplicons size. Barcoded PCR products were cleaned with AMPure beads (REF), the cleaned extracts were checked on a Tapestation bioanalyzer (Agilent) to verify amplicons size, and the final concentration of these extracts was measured using the Qubit fluorometer (Qubit). All samples were diluted to a 4 nano-Molar (nM) concentration and then mixed in equal volumes. Sample preparation and loading onto the sequencer followed the standard Illumina protocol, with the exception of using custom sequencing primers. PhiX control DNA was used for the balanced genome spike to achieve adequate sequence diversity in the run. The prepared samples were sequenced on an Illumina MiSeq sequencer

using a 500 cycle V2 chemistry kit. This kit allows for paired-end 250 bp amplicons to be sequenced.

**After the purchase of primers and completion of sequencing in this study, EMP updated this protocol (http://www.earthmicrobiome.org/protocols-and-standards/16s/ ; updated November 2016) to include new primer sets due to biases in the previous primer set against Crenarachaeota/Thaumarchaeota and SAR11 Alphaproteobacteria (Apprill et al. 2015). These biases are acknowledged in the current study.

References

Apprill, A., McNally, S., Parsons, R., & Webe, L. (2015). Minor revision to V4 region SSU rRNA 806R gene primer greatly increases detection of SAR11 bacterioplankton.

Caporaso, J. G. et al. Ultra-high-throughput microbial community analysis on the Illumina HiSeq and MiSeq platforms. ISME J (2012). doi:10.1038/ismej.2012.8

Caporaso, J. G. et al. Global patterns of 16S rRNA diversity at a depth of millions of sequences per sample. Proc Natl Acad Sci USA 108, 4516–4522 (2011).
