# Supplementary material for: Depth-Dependent Environmental Drivers of Microbial Plankton Community Structure in the Northern Gulf of Mexico
Source: Front Microbiol. 2019 Jan 4;9:3175. doi: 10.3389/fmicb.2018.03175 (PMC6328475; doi:10.3389/fmicb.2018.03175)
Supplement: Supplementary file 10 [file Data_Sheet_2.PDF]

Supplemental Figure 1: Composite chlorophyll a images for DP02. Black dots represent the sampling stations. The background image represents chlorophyll a concentration measured by MODIS aqua and downloaded using NASA's Worldview platform (worldview.earthdata.nasa.gov). Imagery in each panel is a composite of August 9-13 (A), August 14-17 (B), August 18-20 (C), and August 21-14 (D). The white lines in each panel show the cruise track during each imagery date range, except for panel D, which represents a composite of the 4 days after the cruise and the entire cruse track.

Supplemental Figure 2: Inverse Simpson's Index (A) and Faith's Phylogenetic Diversity (B) plotted against sample collection depth. Each color represents a collection station during the cruise DP02.

Supplemental Figure 3: Relative abundance of dominant OTUs across different depths for the seven most abundant taxa in the SRF and EPI (A), the five most abundant taxa in the MESO (B), and the five most abundant taxa in the BATHY (C). Clear differences in dominant taxa across depths can be observed in these plots and corresponds with significant differences in microbial community composition.

Supplemental Figure 4: Relative abundance (proportion of OTU sequences) of dominant taxa (>1% mean relative abundance) in the DCM (A) and BATHY (B) sample depths zones for each station. Taxa are grouped by taxonomy: Act – Actinobacteria, Alp – Alphaproteobacteria, Arc – Archaea, Bac – Bacteroidetes, Cya – Cyanobacteria, Del – Deltaproteobacteria, Euk – microbial eukaryotes, Gam - Gammaproteobacteria, Pla – Planctonycetes.

Supplemental Figure 5: Relative abundance of bacterioplankton phyla (classes for Proteobacteria) for each depth zone in each CTD.

Supplemental Figure 6: CTD profiles for each CTD deployment on DP02. These plots show relationships between Temperature and Depth (A), Density and Depth (B), Salinity and Depth (C), and Temperature and Salinity. Sampling locations on each plot are indicated by colored dots with each unique color representing a unique CTD cast.

Supplemental Figure 7: Relative abundance of six SAR86 Gammaproteobacteria (A), and six Marine Group 1 Thaumarchaeota taxa exhibit shifts in relative abundance across environmental gradients of turbidity (A) and depth (B)

Supplemental Figure 8: Triplot of MESO samples in DP02 that had corresponding nutrient data. Symbols represent unique sampling stations. Vectors in plots represent direction and magnitude of influence for labeled environmental parameter.
