# Supplementary material for: Depth-Dependent Environmental Drivers of Microbial Plankton Community Structure in the Northern Gulf of Mexico
Source: Front Microbiol. 2019 Jan 4;9:3175. doi: 10.3389/fmicb.2018.03175 (PMC6328475; doi:10.3389/fmicb.2018.03175)
Supplement: Supplementary file 11 [file Data_Sheet_3.pdf]

Supplementary File S1: Similarity percentage (SIMPER) analysis results for pairwise microbial plankton differences across pelagic depth zones. Prior to the data, the "Contrast" is displayed and shows the two pelagic depth zones that were compared in the subsequent analysis. The first column shows the microbial taxon ID from the GreenGenes database. The column labeled "average" shows the average abundance of each taxon in the two compared depth zones. The column "sd" displays the standard deviation in taxon abundant, and the column "ratio" shows the ratio of average abundance: standard deviation. Column "ava" shows the average abundance in the first term after "Contrast:" and "avb" shows the average abundance of the second term after "Contrast:" that follows the underscore. Column "cumsum" shows the cumulative variance (difference between two depth zones) explained by the taxa reading the table from top to bottom. Taxa are listed in the order corresponding to their individual explained variance beginning with the taxon that explains the most variance at the top of the table and the least variance at the bottom. Column "p" displays the p-value for each taxon comparison between depth zones based on 999 permutations.

Contrast: Epipelagic_Bathypelagic

	average	sd	ratio	ava	avb	cumsum	p	
355538	3.604e-02	2.605e-02	1.3836	7.221e-02	1.169e-04	0.04007	0.001	***
833419	1.629e-02	7.428e-03	2.1932	6.665e-04	3.325e-02	0.05817	0.001	***
226299	1.583e-02	1.903e-02	0.8318	7.616e-04	3.229e-02	0.07577	0.001	***
777466	1.302e-02	2.999e-02	0.4341	2.408e-02	5.274e-03	0.09025	0.006	**
639502	1.246e-02	6.134e-03	2.0319	2.512e-02	1.946e-04	0.10410	0.001	***
630928	1.218e-02	5.814e-03	2.0951	4.493e-04	2.481e-02	0.11764	0.001	***
557211	1.159e-02	7.029e-03	1.6482	2.505e-02	1.943e-03	0.13052	0.130	
845691	1.125e-02	5.640e-03	1.9941	5.409e-04	2.304e-02	0.14302	0.001	***
427495	1.060e-02	9.083e-03	1.1668	2.205e-02	8.560e-04	0.15480	0.001	***
1080410	1.041e-02	8.360e-03	1.2453	2.152e-03	2.272e-02	0.16637	1.000	
839363	1.008e-02	5.564e-03	1.8126	7.921e-04	2.096e-02	0.17758	0.010	**
341618	9.158e-03	8.482e-03	1.0798	1.840e-02	8.314e-05	0.18776	0.289	
1101488	8.471e-03	2.088e-02	0.4057	1.705e-03	1.779e-02	0.19718	0.067	.
New.ReferenceOTU446	7.458e-03	1.852e-02	0.4027	9.483e-04	1.526e-02	0.20547	0.206	
227663	6.906e-03	4.939e-03	1.3983	1.383e-02	1.531e-05	0.21314	0.001	***
1138756	6.159e-03	1.884e-02	0.3269	1.143e-04	1.230e-02	0.21999	0.118	
228455	6.128e-03	5.747e-03	1.0663	1.228e-02	3.126e-05	0.22680	0.403	
884345	6.102e-03	4.287e-03	1.4233	3.772e-03	1.487e-02	0.23359	0.995	
832816	5.908e-03	3.489e-03	1.6930	1.198e-02	1.666e-04	0.24015	0.001	***
225284	5.646e-03	6.654e-03	0.8485	2.129e-04	1.148e-02	0.24643	0.001	***
694744	5.642e-03	3.774e-03	1.4952	1.129e-02	6.263e-06	0.25270	0.001	***
539147	5.613e-03	2.989e-03	1.8781	1.125e-02	1.890e-05	0.25894	0.001	***
956811	5.608e-03	1.250e-02	0.4488	9.783e-03	2.991e-03	0.26517	0.005	**
1106009	5.514e-03	3.836e-03	1.4374	1.261e-02	1.585e-03	0.27130	0.001	***
833871	5.451e-03	3.253e-03	1.6757	1.092e-02	1.951e-05	0.27736	0.001	***
320821	5.445e-03	3.242e-03	1.6794	1.090e-02	1.107e-05	0.28341	0.001	***
1082059	5.378e-03	3.869e-03	1.3898	7.780e-03	1.373e-02	0.28939	0.488	
1128468	5.024e-03	1.172e-02	0.4288	8.662e-03	2.136e-03	0.29497	0.004	**
1111501	4.851e-03	4.389e-03	1.1052	9.757e-03	5.587e-05	0.30037	0.001	***
351294	4.838e-03	2.845e-03	1.7002	3.754e-03	1.322e-02	0.30574	0.080	.
141607	4.783e-03	4.988e-03	0.9589	3.419e-03	9.380e-03	0.31106	0.001	***

554951	4.765e-03	2.056e-03	2.3181	2.550e-04	9.786e-03	0.31636	0.001	***
231191	4.677e-03	3.023e-03	1.5470	9.753e-03	3.995e-04	0.32156	0.001	***
826517	4.629e-03	1.761e-03	2.6287	3.346e-04	9.592e-03	0.32670	0.001	***
160817	4.594e-03	3.056e-03	1.5035	9.448e-04	1.013e-02	0.33181	0.001	***
4479015	4.537e-03	1.795e-03	2.5273	1.161e-04	9.191e-03	0.33685	0.001	***
509913	4.512e-03	5.564e-03	0.8109	1.238e-03	9.662e-03	0.34187	0.001	***
560276	4.510e-03	4.701e-03	0.9595	9.177e-03	1.797e-04	0.34688	0.001	***
228608	4.104e-03	3.525e-03	1.1643	8.215e-03	5.633e-06	0.35144	0.001	***
753560	3.856e-03	6.743e-03	0.5718	7.390e-04	8.273e-03	0.35573	0.047	*
268433	3.815e-03	2.093e-03	1.8224	7.685e-03	7.102e-05	0.35997	0.001	***
315098	3.660e-03	2.955e-03	1.2387	7.451e-03	1.726e-04	0.36404	0.001	***
358464	3.549e-03	9.700e-03	0.3659	2.067e-05	7.086e-03	0.36798	0.078	.
549048	3.516e-03	3.796e-03	0.9263	7.045e-03	1.624e-05	0.37189	0.001	***
592779	3.410e-03	3.164e-03	1.0780	6.824e-03	4.663e-06	0.37568	0.001	***
834169	3.398e-03	1.818e-03	1.8695	1.207e-05	6.808e-03	0.37946	0.001	***
New.ReferenceOTU128	3.274e-03	1.200e-03	2.7291	3.681e-04	6.916e-03	0.38310	0.001	***
1007856	3.232e-03	1.369e-03	2.3609	1.449e-04	6.601e-03	0.38669	0.001	***
310272	3.185e-03	2.373e-03	1.3424	3.581e-04	6.705e-03	0.39023	0.696	
250570	3.182e-03	2.523e-03	1.2611	6.862e-03	6.452e-04	0.39377	0.001	***
554945	3.175e-03	2.232e-03	1.4227	6.351e-03	0.000e+00	0.39730	0.001	***
1108577	3.150e-03	3.541e-03	0.8896	6.383e-03	8.373e-05	0.40080	0.789	
60638	3.136e-03	2.971e-03	1.0558	6.273e-03	0.000e+00	0.40429	0.001	***
323489	3.077e-03	3.040e-03	1.0123	6.590e-04	6.612e-03	0.40771	1.000	
711803	3.001e-03	3.963e-03	0.7572	3.467e-06	6.006e-03	0.41104	0.001	***
New.ReferenceOTU698	2.994e-03	1.106e-03	2.7060	6.008e-03	2.000e-05	0.41437	0.001	***
646549	2.840e-03	3.667e-03	0.7747	1.752e-03	6.182e-03	0.41753	0.810	
119502	2.801e-03	2.793e-03	1.0028	5.738e-03	1.612e-04	0.42064	0.001	***
1107076	2.735e-03	4.074e-03	0.6713	3.017e-03	4.118e-03	0.42368	0.209	
839203	2.679e-03	1.527e-03	1.7538	7.634e-06	5.365e-03	0.42666	0.001	***
536001	2.676e-03	3.400e-03	0.7870	5.700e-03	5.022e-04	0.42963	0.231	
New.ReferenceOTU882	2.668e-03	2.832e-03	0.9422	5.379e-03	4.306e-05	0.43260	0.514	
844663	2.635e-03	1.590e-03	1.6575	2.199e-04	5.465e-03	0.43553	0.001	***
110119	2.635e-03	1.818e-03	1.4488	5.074e-03	8.588e-03	0.43846	0.161	
581028	2.572e-03	1.713e-03	1.5015	5.844e-04	5.717e-03	0.44132	0.001	***
860929	2.544e-03	2.994e-03	0.8498	4.891e-04	5.299e-03	0.44414	0.001	***

New.ReferenceOTU44	2.482e-03	2.116e-03	1.1731	5.522e-04	5.494e-03	0.44690	0.551	
842800	2.455e-03	1.541e-03	1.5928	2.461e-04	5.150e-03	0.44963	0.987	
817600	2.413e-03	2.129e-03	1.1338	4.879e-03	5.665e-05	0.45231	1.000	
352531	2.382e-03	2.875e-03	0.8285	4.769e-03	5.535e-06	0.45496	0.578	
1105428	2.369e-03	3.771e-03	0.6281	4.742e-03	6.344e-06	0.45759	0.820	
833961	2.351e-03	1.954e-03	1.2031	4.718e-03	1.600e-05	0.46021	0.001	***
103709	2.314e-03	8.228e-04	2.8124	4.652e-03	2.347e-05	0.46278	0.001	***
3156804	2.276e-03	1.248e-03	1.8245	4.688e-03	1.620e-04	0.46531	0.005	**
832702	2.276e-03	8.434e-04	2.6983	6.808e-05	4.619e-03	0.46784	0.001	***
979107	2.265e-03	4.261e-03	0.5315	4.548e-04	4.800e-03	0.47036	0.058	.
834856	2.186e-03	1.256e-03	1.7403	1.844e-04	4.546e-03	0.47279	0.001	***
353181	2.135e-03	1.539e-03	1.3868	4.309e-03	3.941e-05	0.47516	0.579	
361839	2.131e-03	1.562e-03	1.3648	4.314e-03	5.215e-05	0.47753	0.001	***
New.ReferenceOTU397	2.108e-03	1.735e-03	1.2150	4.215e-03	0.000e+00	0.47987	0.001	***
832374	2.107e-03	9.101e-04	2.3153	2.119e-04	4.411e-03	0.48221	0.001	***
New.ReferenceOTU697	2.098e-03	7.461e-04	2.8118	7.883e-05	4.275e-03	0.48455	0.001	***
810040	2.048e-03	9.267e-04	2.2099	2.945e-05	4.125e-03	0.48682	0.001	***
542823	2.047e-03	1.970e-03	1.0389	4.143e-03	5.139e-05	0.48910	0.003	**
New.ReferenceOTU414	2.025e-03	2.051e-03	0.9872	5.024e-04	4.462e-03	0.49135	0.001	***
653733	1.981e-03	1.659e-03	1.1941	3.968e-03	5.712e-06	0.49355	0.001	***
830634	1.937e-03	1.308e-03	1.4807	6.178e-05	3.931e-03	0.49570	0.002	**
New.ReferenceOTU610	1.934e-03	2.302e-03	0.8402	3.873e-03	8.706e-06	0.49785	0.001	***
544313	1.907e-03	2.287e-03	0.8336	1.426e-04	3.896e-03	0.49997	0.003	**
838079	1.888e-03	1.015e-03	1.8600	1.270e-05	3.787e-03	0.50207	0.001	***
119137	1.886e-03	2.273e-03	0.8297	4.694e-03	1.326e-03	0.50417	0.005	**
1102212	1.878e-03	1.377e-03	1.3640	3.757e-03	0.000e+00	0.50626	0.001	***
147658	1.865e-03	1.405e-03	1.3276	3.887e-03	1.982e-04	0.50833	0.001	***
548576	1.854e-03	3.531e-03	0.5252	1.827e-04	3.759e-03	0.51039	0.019	*
New.ReferenceOTU71	1.848e-03	1.299e-03	1.4220	3.700e-03	6.332e-06	0.51244	0.001	***
581343	1.837e-03	8.700e-04	2.1117	3.779e-03	1.043e-04	0.51448	0.001	***
182418	1.810e-03	3.928e-03	0.4607	2.546e-04	3.626e-03	0.51650	0.064	.
108953	1.785e-03	8.113e-04	2.2000	5.980e-05	3.629e-03	0.51848	0.001	***
347913	1.782e-03	1.152e-03	1.5462	2.250e-04	3.779e-03	0.52046	0.388	
592269	1.779e-03	3.056e-03	0.5821	3.643e-03	9.686e-05	0.52244	0.990	
4479541	1.767e-03	1.288e-03	1.3722	3.540e-03	6.612e-06	0.52440	0.001	***

```
834104                         1.765e-03 8.755e-04 2.0161 2.118e-04 3.732e-03 0.52636 0.001 ***
1084865                        1.749e-03 1.881e-03 0.9300 5.411e-04 3.979e-03 0.52831 0.002 **
583907                         1.738e-03 4.468e-03 0.3889 2.741e-05 3.467e-03 0.53024 0.083 .
234197                         1.733e-03 1.353e-03 1.2813 3.644e-03 2.186e-04 0.53217 0.001 ***
826571                         1.729e-03 7.744e-04 2.2325 6.426e-05 3.522e-03 0.53409 0.001 ***
833273                         1.727e-03 9.087e-04 1.9004 2.036e-04 3.657e-03 0.53601 0.001 ***
355527                         1.701e-03 2.819e-03 0.6032 3.442e-03 4.734e-05 0.53790 0.999
112306                         1.691e-03 9.739e-04 1.7358 2.970e-05 3.408e-03 0.53978 0.001 ***
757981                         1.683e-03 9.428e-04 1.7853 1.615e-05 3.382e-03 0.54165 0.001 ***
New.ReferenceOTU752            1.668e-03 4.837e-03 0.3449 5.765e-04 3.229e-03 0.54350 0.216
552671                         1.658e-03 2.221e-03 0.7467 7.182e-05 3.380e-03 0.54535 0.001 ***
926160                         1.652e-03 8.630e-03 0.1915 7.917e-05 3.274e-03 0.54718 0.343
1584736                        1.642e-03 1.176e-03 1.3969 3.285e-03 1.772e-07 0.54901 0.001 ***
823035                         1.630e-03 2.082e-03 0.7829 3.478e-03 3.471e-04 0.55082 0.001 ***
779725                         1.615e-03 1.004e-03 1.6089 4.592e-04 3.613e-03 0.55262 0.001 ***
825320                         1.578e-03 1.910e-03 0.8265 3.220e-03 8.876e-05 0.55437 0.001 ***
317124                         1.576e-03 4.423e-03 0.3564 3.148e-03 8.989e-06 0.55612 0.004 **
231973                         1.572e-03 2.246e-03 0.6999 3.165e-03 3.957e-05 0.55787 0.001 ***
836341                         1.566e-03 1.031e-03 1.5183 1.146e-03 3.798e-03 0.55961 0.001 ***
227717                         1.554e-03 1.394e-03 1.1146 3.131e-03 2.473e-05 0.56134 0.002 **
827407                         1.550e-03 9.002e-04 1.7217 7.352e-04 3.790e-03 0.56306 0.011 *
830400                         1.522e-03 1.352e-03 1.1256 3.473e-03 9.216e-04 0.56475 0.001 ***
1105105                        1.522e-03 7.978e-04 1.9076 3.180e-03 1.365e-04 0.56644 0.104
841316                         1.517e-03 8.065e-04 1.8806 6.043e-04 3.602e-03 0.56813 0.001 ***
 [ reached getOption("max.print") -- omitted 7857 rows ]

Contrast: Epipelagic_Surface

                                 average        sd  ratio       ava       avb  cumsum     p
355538                         2.931e-02 2.346e-02 1.2494 7.221e-02 5.953e-02 0.04606 0.005 **
557211                         1.325e-02 9.795e-03 1.3527 2.505e-02 4.150e-02 0.06688 0.013 *
777466                         1.206e-02 3.087e-02 0.3906 2.408e-02 1.433e-03 0.08583 0.050 *
817600                         1.204e-02 1.110e-02 1.0850 4.879e-03 2.864e-02 0.10475 0.001 ***
341618                         1.196e-02 7.224e-03 1.6556 1.840e-02 3.711e-02 0.12354 0.002 **
228455                         9.238e-03 1.086e-02 0.8505 1.228e-02 2.176e-02 0.13806 0.002 **
```

639502	8.740e-03	7.036e-03	1.2423	2.512e-02	3.588e-02	0.15179	0.630	
427495	8.172e-03	7.645e-03	1.0691	2.205e-02	2.029e-02	0.16464	0.045	*
355527	7.999e-03	7.910e-03	1.0113	3.442e-03	1.774e-02	0.17721	0.001	***
315563	7.553e-03	1.109e-02	0.6812	1.147e-03	1.501e-02	0.18908	0.001	***
1082059	6.900e-03	9.401e-03	0.7339	7.780e-03	1.195e-02	0.19992	0.035	*
1108577	5.623e-03	3.723e-03	1.5104	6.383e-03	1.501e-02	0.20876	0.001	***
592269	5.426e-03	3.458e-03	1.5691	3.643e-03	1.256e-02	0.21728	0.001	***
227663	5.267e-03	4.319e-03	1.2194	1.383e-02	7.264e-03	0.22556	0.001	***
694744	5.221e-03	3.634e-03	1.4368	1.129e-02	1.245e-03	0.23376	0.001	***
1105428	5.127e-03	5.070e-03	1.0112	4.742e-03	1.106e-02	0.24182	0.001	***
956811	4.969e-03	1.296e-02	0.3834	9.783e-03	8.836e-04	0.24963	0.054	.
833871	4.953e-03	3.147e-03	1.5736	1.092e-02	2.102e-03	0.25741	0.001	***
539147	4.845e-03	2.794e-03	1.7342	1.125e-02	3.961e-03	0.26503	0.001	***
320821	4.599e-03	3.075e-03	1.4954	1.090e-02	2.987e-03	0.27225	0.001	***
1109816	4.551e-03	3.350e-03	1.3583	2.097e-03	1.076e-02	0.27940	0.001	***
1128468	4.357e-03	1.213e-02	0.3593	8.662e-03	1.694e-04	0.28625	0.055	.
592771	4.275e-03	2.744e-03	1.5579	2.233e-03	9.343e-03	0.29297	0.001	***
New.ReferenceOTU882	4.212e-03	3.967e-03	1.0616	5.379e-03	1.050e-02	0.29959	0.001	***
151344	4.065e-03	4.034e-03	1.0078	2.807e-04	8.360e-03	0.30598	0.001	***
560276	4.063e-03	4.499e-03	0.9031	9.177e-03	1.875e-03	0.31236	0.001	***
536001	4.050e-03	3.739e-03	1.0830	5.700e-03	7.657e-03	0.31872	0.001	***
352531	4.044e-03	3.314e-03	1.2205	4.769e-03	9.698e-03	0.32508	0.001	***
1111501	3.986e-03	3.202e-03	1.2448	9.757e-03	1.046e-02	0.33134	0.029	*
231191	3.954e-03	3.348e-03	1.1809	9.753e-03	8.234e-03	0.33756	0.003	**
341734	3.844e-03	2.672e-03	1.4389	8.622e-04	8.276e-03	0.34360	0.001	***
228608	3.824e-03	3.296e-03	1.1602	8.215e-03	1.919e-03	0.34961	0.001	***
338106	3.688e-03	5.263e-03	0.7008	2.694e-04	7.414e-03	0.35540	0.001	***
343231	3.667e-03	3.042e-03	1.2054	2.305e-03	8.194e-03	0.36117	0.001	***
1106009	3.551e-03	2.839e-03	1.2507	1.261e-02	1.295e-02	0.36675	0.422	
268433	3.491e-03	2.043e-03	1.7083	7.685e-03	1.157e-03	0.37223	0.001	***
106179	3.447e-03	1.976e-03	1.7442	1.701e-03	8.257e-03	0.37765	0.001	***
315098	3.438e-03	2.858e-03	1.2028	7.451e-03	9.621e-04	0.38305	0.001	***
549048	3.418e-03	3.683e-03	0.9280	7.045e-03	5.514e-04	0.38842	0.001	***
832816	3.358e-03	2.685e-03	1.2510	1.198e-02	9.769e-03	0.39370	0.401	
592779	3.207e-03	3.064e-03	1.0468	6.824e-03	7.887e-04	0.39874	0.001	***

250570	3.111e-03	2.446e-03	1.2721	6.862e-03	1.694e-03	0.40363	0.001	***
353181	3.030e-03	2.228e-03	1.3599	4.309e-03	9.260e-03	0.40839	0.001	***
554945	2.967e-03	2.144e-03	1.3837	6.351e-03	1.121e-03	0.41305	0.001	***
119502	2.780e-03	2.693e-03	1.0324	5.738e-03	9.477e-04	0.41742	0.001	***
60638	2.749e-03	2.841e-03	0.9677	6.273e-03	1.380e-03	0.42174	0.001	***
551207	2.500e-03	2.838e-03	0.8808	1.362e-03	5.539e-03	0.42567	0.001	***
740763	2.497e-03	2.340e-03	1.0672	1.383e-03	5.397e-03	0.42960	0.001	***
110119	2.315e-03	1.850e-03	1.2516	5.074e-03	1.083e-03	0.43323	0.902	
774258	2.292e-03	2.937e-03	0.7804	7.779e-04	4.616e-03	0.43683	0.001	***
234994	2.290e-03	2.139e-03	1.0706	1.263e-03	4.802e-03	0.44043	0.001	***
833961	2.217e-03	1.890e-03	1.1728	4.718e-03	1.154e-03	0.44392	0.001	***
773487	2.185e-03	1.359e-03	1.6077	7.327e-04	4.799e-03	0.44735	0.001	***
119137	2.160e-03	1.856e-03	1.1634	4.694e-03	3.876e-03	0.45074	0.001	***
1107422	2.130e-03	2.883e-03	0.7390	8.287e-04	4.023e-03	0.45409	0.001	***
591130	2.115e-03	2.465e-03	0.8580	1.281e-03	5.109e-03	0.45742	0.001	***
234927	2.115e-03	1.397e-03	1.5131	1.749e-03	5.574e-03	0.46074	0.001	***
884345	2.080e-03	2.664e-03	0.7808	3.772e-03	1.926e-03	0.46401	1.000	
147658	2.012e-03	1.523e-03	1.3210	3.887e-03	1.212e-03	0.46717	0.001	***
New.ReferenceOTU252	2.006e-03	3.001e-03	0.6685	8.859e-05	3.987e-03	0.47032	0.001	***
New.ReferenceOTU397	1.979e-03	1.685e-03	1.1745	4.215e-03	4.020e-04	0.47343	0.001	***
3156804	1.957e-03	1.645e-03	1.1898	4.688e-03	6.879e-03	0.47651	0.178	
232905	1.953e-03	1.132e-03	1.7252	9.775e-04	4.573e-03	0.47958	0.001	***
314474	1.946e-03	1.253e-03	1.5531	5.172e-04	4.322e-03	0.48263	0.001	***
592350	1.888e-03	2.229e-03	0.8470	2.360e-03	5.104e-03	0.48560	0.002	**
New.ReferenceOTU610	1.883e-03	2.232e-03	0.8435	3.873e-03	3.240e-04	0.48856	0.001	***
361839	1.867e-03	1.488e-03	1.2551	4.314e-03	9.049e-04	0.49149	0.001	***
4475220	1.847e-03	1.594e-03	1.1586	6.060e-04	4.066e-03	0.49440	0.001	***
542823	1.837e-03	1.608e-03	1.1425	4.143e-03	5.397e-03	0.49728	0.042	*
227866	1.827e-03	1.899e-03	0.9618	1.386e-03	3.985e-03	0.50015	0.001	***
1102212	1.825e-03	1.340e-03	1.3612	3.757e-03	9.821e-04	0.50302	0.001	***
653733	1.770e-03	1.576e-03	1.1228	3.968e-03	7.598e-04	0.50580	0.001	***
356368	1.740e-03	9.645e-04	1.8046	4.898e-04	3.878e-03	0.50854	0.001	***
243160	1.739e-03	1.737e-03	1.0013	1.441e-04	3.596e-03	0.51127	0.001	***
1108696	1.730e-03	1.291e-03	1.3400	5.208e-04	3.868e-03	0.51399	0.001	***
563843	1.704e-03	1.298e-03	1.3132	2.919e-03	5.795e-03	0.51667	0.015	*

1109402	1.671e-03	1.369e-03	1.2206	6.559e-04	3.585e-03	0.51929	0.001	***
141607	1.661e-03	3.741e-03	0.4439	3.419e-03	6.523e-04	0.52190	0.995	
823035	1.658e-03	2.076e-03	0.7987	3.478e-03	3.427e-04	0.52451	0.001	***
830400	1.643e-03	1.472e-03	1.1164	3.473e-03	3.754e-04	0.52709	0.001	***
1105105	1.612e-03	1.157e-03	1.3929	3.180e-03	5.839e-03	0.52962	0.040	*
249226	1.606e-03	1.023e-03	1.5695	1.587e-03	4.293e-03	0.53215	0.001	***
569884	1.605e-03	1.499e-03	1.0709	1.484e-03	3.815e-03	0.53467	0.001	***
4479541	1.604e-03	1.237e-03	1.2971	3.540e-03	6.244e-04	0.53719	0.001	***
317124	1.598e-03	4.405e-03	0.3629	3.148e-03	7.802e-05	0.53970	0.001	***
323305	1.595e-03	2.221e-03	0.7182	5.686e-04	3.273e-03	0.54221	0.001	***
234197	1.591e-03	1.284e-03	1.2396	3.644e-03	6.669e-04	0.54471	0.001	***
New.ReferenceOTU238	1.590e-03	1.062e-03	1.4970	1.487e-03	4.406e-03	0.54721	0.001	***
344865	1.559e-03	8.783e-04	1.7752	8.894e-04	3.706e-03	0.54966	0.001	***
344629	1.557e-03	1.455e-03	1.0698	9.589e-04	3.378e-03	0.55210	0.001	***
1584736	1.538e-03	1.142e-03	1.3464	3.285e-03	3.070e-04	0.55452	0.001	***
1011088	1.536e-03	1.852e-03	0.8294	1.784e-04	3.224e-03	0.55693	0.074	.
New.ReferenceOTU698	1.528e-03	1.207e-03	1.2664	6.008e-03	3.668e-03	0.55934	0.261	
New.ReferenceOTU71	1.524e-03	1.072e-03	1.4215	3.700e-03	2.081e-03	0.56173	0.001	***
825320	1.507e-03	1.881e-03	0.8014	3.220e-03	3.630e-04	0.56410	0.001	***
245143	1.500e-03	1.926e-03	0.7786	5.752e-04	2.856e-03	0.56646	0.001	***
899488	1.491e-03	2.292e-03	0.6505	2.232e-03	1.304e-03	0.56880	0.001	***
1107076	1.481e-03	1.390e-03	1.0652	3.017e-03	8.876e-04	0.57113	0.984	
351294	1.477e-03	1.150e-03	1.2849	3.754e-03	1.923e-03	0.57345	1.000	
231973	1.431e-03	2.125e-03	0.6737	3.165e-03	7.665e-04	0.57570	0.001	***
579841	1.407e-03	1.056e-03	1.3318	2.934e-03	3.549e-03	0.57791	0.002	**
2138725	1.397e-03	1.499e-03	0.9318	2.938e-03	3.445e-04	0.58010	0.001	***
575540	1.396e-03	1.154e-03	1.2093	1.805e-04	2.949e-03	0.58230	0.001	***
929908	1.393e-03	1.614e-03	0.8632	2.557e-03	2.703e-03	0.58449	0.002	**
548737	1.393e-03	1.786e-03	0.7799	1.415e-03	2.322e-03	0.58668	0.001	***
147786	1.384e-03	1.256e-03	1.1024	2.435e-03	3.066e-03	0.58885	0.001	***
581343	1.338e-03	8.631e-04	1.5504	3.779e-03	1.481e-03	0.59096	0.001	***
1080410	1.335e-03	2.159e-03	0.6184	2.152e-03	1.865e-03	0.59305	1.000	
825365	1.319e-03	1.675e-03	0.7876	1.904e-03	1.191e-03	0.59513	0.001	***
4160085	1.313e-03	2.107e-03	0.6232	1.205e-03	2.472e-03	0.59719	0.002	**
585911	1.293e-03	9.779e-04	1.3221	4.194e-04	2.811e-03	0.59922	0.001	***

```
227717                        1.289e-03 9.738e-04 1.3241 3.131e-03 3.492e-03 0.60125 0.024 *
354725                        1.261e-03 6.597e-04 1.9113 8.373e-04 3.170e-03 0.60323 0.001 ***
New.ReferenceOTU787           1.259e-03 1.144e-03 1.1010 2.259e-03 1.067e-03 0.60521 0.001 ***
264786                        1.246e-03 2.074e-03 0.6007 7.107e-05 2.529e-03 0.60717 0.001 ***
551347                        1.241e-03 9.150e-04 1.3559 1.029e-03 2.719e-03 0.60912 0.001 ***
848082                        1.212e-03 1.555e-03 0.7793 6.694e-04 2.387e-03 0.61102 0.001 ***
New.ReferenceOTU399           1.211e-03 8.619e-04 1.4046 2.076e-03 4.131e-03 0.61292 0.005 **
846727                        1.203e-03 1.022e-03 1.1771 2.555e-03 3.506e-04 0.61481 0.994
179922                        1.174e-03 8.504e-04 1.3803 7.055e-04 2.423e-03 0.61666 0.001 ***
New.ReferenceOTU874           1.171e-03 1.315e-03 0.8905 2.451e-03 2.759e-04 0.61850 0.001 ***
1543166                       1.160e-03 9.415e-04 1.2325 1.617e-03 2.283e-03 0.62032 0.001 ***
137211                        1.159e-03 9.838e-04 1.1783 2.761e-03 8.139e-04 0.62214 0.001 ***
349161                        1.158e-03 7.273e-04 1.5916 1.009e-03 2.715e-03 0.62396 0.001 ***
229118                        1.143e-03 8.278e-04 1.3808 2.091e-03 3.207e-03 0.62576 0.004 **
 [ reached getOption("max.print") -- omitted 7857 rows ]

Contrast: Epipelagic_Mesopelagic

                                average        sd  ratio       ava       avb  cumsum     p
1080410                       3.471e-02 1.593e-02 2.1785 2.152e-03 7.157e-02 0.04076 0.001 ***
355538                        3.440e-02 2.595e-02 1.3257 7.221e-02 3.879e-03 0.08115 0.001 ***
323489                        1.979e-02 1.313e-02 1.5074 6.590e-04 4.023e-02 0.10439 0.001 ***
34469                         1.677e-02 7.751e-03 2.1642 7.590e-04 3.431e-02 0.12408 0.001 ***
839363                        1.469e-02 7.123e-03 2.0623 7.921e-04 3.017e-02 0.14133 0.001 ***
884345                        1.378e-02 7.603e-03 1.8132 3.772e-03 3.115e-02 0.15752 0.001 ***
777466                        1.364e-02 2.954e-02 0.4616 2.408e-02 7.329e-03 0.17353 0.004 **
639502                        1.119e-02 6.436e-03 1.7381 2.512e-02 2.811e-03 0.18667 0.004 **
557211                        1.096e-02 7.025e-03 1.5601 2.505e-02 3.492e-03 0.19954 0.312
427495                        1.042e-02 9.081e-03 1.1474 2.205e-02 1.237e-03 0.21177 0.001 ***
833419                        9.663e-03 4.055e-03 2.3831 6.665e-04 1.999e-02 0.22312 0.175
341618                        8.676e-03 8.497e-03 1.0211 1.840e-02 1.082e-03 0.23330 0.493
845691                        7.528e-03 4.374e-03 1.7212 5.409e-04 1.560e-02 0.24214 0.044 *
351294                        6.962e-03 3.074e-03 2.2650 3.754e-03 1.762e-02 0.25032 0.001 ***
630928                        6.845e-03 3.383e-03 2.0236 4.493e-04 1.414e-02 0.25836 0.420
108965                        6.620e-03 3.477e-03 1.9041 3.714e-04 1.361e-02 0.26613 0.001 ***
```

227663	6.491e-03	4.901e-03	1.3243	1.383e-02	1.079e-03	0.27375	0.001	***
228455	6.023e-03	5.713e-03	1.0542	1.228e-02	3.306e-04	0.28082	0.451	
310272	5.752e-03	2.797e-03	2.0566	3.581e-04	1.186e-02	0.28758	0.001	***
832816	5.656e-03	3.489e-03	1.6213	1.198e-02	7.112e-04	0.29422	0.001	***
842800	5.609e-03	3.083e-03	1.8195	2.461e-04	1.146e-02	0.30081	0.001	***
694744	5.575e-03	3.749e-03	1.4869	1.129e-02	1.926e-04	0.30735	0.001	***
956811	5.461e-03	1.263e-02	0.4323	9.783e-03	2.568e-03	0.31377	0.006	**
539147	5.276e-03	2.916e-03	1.8091	1.125e-02	8.619e-04	0.31996	0.001	***
320821	5.273e-03	3.221e-03	1.6368	1.090e-02	4.113e-04	0.32615	0.001	***
833871	5.148e-03	3.160e-03	1.6290	1.092e-02	8.267e-04	0.33220	0.001	***
1106009	4.875e-03	3.863e-03	1.2620	1.261e-02	2.894e-03	0.33792	0.001	***
1128468	4.720e-03	1.188e-02	0.3974	8.662e-03	1.317e-03	0.34347	0.009	**
1111501	4.504e-03	4.353e-03	1.0347	9.757e-03	9.192e-04	0.34876	0.001	***
646549	4.418e-03	6.371e-03	0.6935	1.752e-03	9.228e-03	0.35394	0.079	.
231191	4.304e-03	2.993e-03	1.4383	9.753e-03	1.200e-03	0.35900	0.001	***
560276	3.875e-03	4.534e-03	0.8548	9.177e-03	1.953e-03	0.36355	0.001	***
228608	3.810e-03	3.387e-03	1.1249	8.215e-03	9.005e-04	0.36802	0.001	***
268433	3.761e-03	2.074e-03	1.8137	7.685e-03	2.199e-04	0.37244	0.001	***
125125	3.635e-03	1.386e-03	2.6225	3.956e-04	7.662e-03	0.37671	0.001	***
New.ReferenceOTU446	3.592e-03	6.469e-03	0.5553	9.483e-04	7.663e-03	0.38092	0.876	
826517	3.534e-03	1.524e-03	2.3195	3.346e-04	7.403e-03	0.38507	0.001	***
549048	3.505e-03	3.792e-03	0.9243	7.045e-03	5.023e-05	0.38919	0.001	***
250570	3.405e-03	2.605e-03	1.3071	6.862e-03	5.750e-05	0.39319	0.001	***
837317	3.369e-03	2.095e-03	1.6083	3.652e-04	7.094e-03	0.39714	0.001	***
592779	3.352e-03	3.128e-03	1.0717	6.824e-03	2.909e-04	0.40108	0.001	***
1082059	3.342e-03	3.614e-03	0.9248	7.780e-03	6.702e-03	0.40500	0.999	
New.ReferenceOTU44	3.218e-03	5.739e-03	0.5608	5.522e-04	6.929e-03	0.40878	0.129	
554945	3.130e-03	2.218e-03	1.4115	6.351e-03	1.367e-04	0.41246	0.001	***
347913	3.128e-03	1.246e-03	2.5098	2.250e-04	6.481e-03	0.41613	0.001	***
60638	3.105e-03	2.967e-03	1.0465	6.273e-03	7.284e-05	0.41978	0.001	***
1108577	3.084e-03	3.543e-03	0.8704	6.383e-03	2.150e-04	0.42340	0.808	
325965	2.990e-03	1.292e-03	2.3146	2.416e-04	6.220e-03	0.42691	0.001	***
832374	2.928e-03	1.023e-03	2.8630	2.119e-04	6.062e-03	0.43035	0.001	***
New.ReferenceOTU698	2.872e-03	1.137e-03	2.5248	6.008e-03	2.650e-04	0.43372	0.001	***
834856	2.866e-03	9.784e-04	2.9295	1.844e-04	5.917e-03	0.43709	0.001	***

315098	2.792e-03	2.140e-03	1.3042	7.451e-03	5.723e-03	0.44036	0.005	**
154513	2.719e-03	2.047e-03	1.3282	3.253e-04	5.746e-03	0.44356	0.001	***
536001	2.614e-03	3.361e-03	0.7776	5.700e-03	7.741e-04	0.44663	0.308	
New.ReferenceOTU882	2.581e-03	2.829e-03	0.9122	5.379e-03	2.413e-04	0.44966	0.600	
826533	2.551e-03	5.780e-03	0.4414	7.708e-04	4.987e-03	0.45265	0.127	
837714	2.439e-03	1.007e-03	2.4207	9.407e-04	5.786e-03	0.45552	0.001	***
825985	2.417e-03	1.018e-03	2.3736	3.223e-04	5.150e-03	0.45835	0.001	***
110119	2.412e-03	1.668e-03	1.4463	5.074e-03	8.296e-03	0.46119	0.741	
1105428	2.344e-03	3.739e-03	0.6268	4.742e-03	2.092e-04	0.46394	0.824	
817600	2.338e-03	2.100e-03	1.1135	4.879e-03	2.826e-04	0.46668	1.000	
119502	2.334e-03	2.567e-03	0.9092	5.738e-03	1.743e-03	0.46942	0.001	***
352531	2.327e-03	2.864e-03	0.8125	4.769e-03	1.493e-04	0.47216	0.653	
554951	2.326e-03	1.189e-03	1.9561	2.550e-04	4.902e-03	0.47489	0.919	
1007856	2.323e-03	8.298e-04	2.7992	1.449e-04	4.783e-03	0.47762	0.001	***
New.ReferenceOTU421	2.305e-03	7.257e-04	3.1760	2.713e-04	4.881e-03	0.48032	0.001	***
831163	2.276e-03	1.425e-03	1.5974	4.096e-04	4.951e-03	0.48300	0.001	***
833961	2.271e-03	1.932e-03	1.1756	4.718e-03	2.810e-04	0.48566	0.001	***
573845	2.260e-03	4.851e-03	0.4659	2.874e-05	4.528e-03	0.48832	0.138	
827407	2.241e-03	9.047e-04	2.4767	7.352e-04	5.217e-03	0.49095	0.001	***
141607	2.230e-03	3.718e-03	0.5999	3.419e-03	2.648e-03	0.49357	0.945	
3156804	2.143e-03	1.260e-03	1.7000	4.688e-03	4.141e-04	0.49608	0.024	*
103709	2.127e-03	8.633e-04	2.4633	4.652e-03	4.817e-04	0.49858	0.001	***
253327	2.112e-03	2.971e-03	0.7108	1.484e-03	4.478e-03	0.50106	0.013	*
347857	2.107e-03	9.338e-04	2.2566	1.014e-04	4.310e-03	0.50353	0.001	***
119137	2.091e-03	2.374e-03	0.8807	4.694e-03	5.858e-04	0.50599	0.001	***
361839	2.078e-03	1.557e-03	1.3343	4.314e-03	1.745e-04	0.50843	0.001	***
New.ReferenceOTU397	2.074e-03	1.722e-03	1.2042	4.215e-03	1.003e-04	0.51086	0.001	***
353181	2.020e-03	1.520e-03	1.3285	4.309e-03	4.774e-04	0.51324	0.755	
1107076	1.979e-03	1.433e-03	1.3811	3.017e-03	5.312e-03	0.51556	0.817	
New.ReferenceOTU610	1.930e-03	2.299e-03	0.8397	3.873e-03	2.500e-05	0.51783	0.001	***
653733	1.927e-03	1.649e-03	1.1680	3.968e-03	1.507e-04	0.52009	0.001	***
542823	1.915e-03	1.953e-03	0.9801	4.143e-03	3.862e-04	0.52234	0.009	**
825733	1.853e-03	9.637e-04	1.9226	1.365e-03	4.945e-03	0.52451	0.001	***
1102212	1.845e-03	1.361e-03	1.3561	3.757e-03	9.797e-05	0.52668	0.001	***
834286	1.825e-03	9.359e-04	1.9502	6.925e-05	3.720e-03	0.52882	0.001	***

161219	1.803e-03	8.250e-04	2.1855	9.167e-05	3.692e-03	0.53094	0.001	***
592269	1.797e-03	3.035e-03	0.5921	3.643e-03	2.179e-04	0.53305	0.991	
New.ReferenceOTU71	1.786e-03	1.270e-03	1.4063	3.700e-03	2.226e-04	0.53515	0.001	***
581343	1.752e-03	8.849e-04	1.9803	3.779e-03	3.192e-04	0.53720	0.001	***
4479541	1.740e-03	1.282e-03	1.3574	3.540e-03	7.409e-05	0.53925	0.001	***
1101488	1.735e-03	2.140e-03	0.8103	1.705e-03	3.782e-03	0.54128	0.965	
823035	1.711e-03	2.103e-03	0.8134	3.478e-03	1.460e-04	0.54329	0.001	***
355527	1.668e-03	2.809e-03	0.5939	3.442e-03	1.656e-04	0.54525	0.997	
101670	1.666e-03	9.501e-04	1.7536	6.366e-04	3.872e-03	0.54721	0.001	***
581028	1.662e-03	9.841e-04	1.6887	5.844e-04	3.866e-03	0.54916	0.417	
834243	1.648e-03	1.715e-03	0.9609	1.667e-04	3.398e-03	0.55110	0.001	***
1584736	1.632e-03	1.172e-03	1.3925	3.285e-03	2.652e-05	0.55301	0.001	***
830634	1.630e-03	1.883e-03	0.8659	6.178e-05	3.309e-03	0.55493	0.182	
1074804	1.623e-03	7.755e-04	2.0929	1.160e-04	3.362e-03	0.55683	0.001	***
234197	1.621e-03	1.299e-03	1.2483	3.644e-03	5.968e-04	0.55874	0.001	***
147658	1.612e-03	1.291e-03	1.2479	3.887e-03	9.641e-04	0.56063	0.001	***
6258	1.603e-03	1.038e-03	1.5448	1.510e-03	4.325e-03	0.56251	0.001	***
846727	1.595e-03	1.177e-03	1.3551	2.555e-03	4.824e-03	0.56438	0.041	*
317124	1.574e-03	4.425e-03	0.3557	3.148e-03	8.706e-07	0.56623	0.004	**
231973	1.573e-03	2.246e-03	0.7005	3.165e-03	3.556e-05	0.56808	0.001	***
830400	1.567e-03	1.418e-03	1.1052	3.473e-03	6.134e-04	0.56992	0.001	***
838996	1.517e-03	8.355e-04	1.8151	2.805e-05	3.061e-03	0.57170	0.001	***
753560	1.475e-03	2.092e-03	0.7051	7.390e-04	3.340e-03	0.57343	0.972	
227717	1.474e-03	1.373e-03	1.0741	3.131e-03	2.750e-04	0.57516	0.001	***
825320	1.442e-03	1.863e-03	0.7742	3.220e-03	5.119e-04	0.57686	0.001	***
1105105	1.432e-03	8.111e-04	1.7648	3.180e-03	3.208e-04	0.57854	0.295	
834104	1.429e-03	7.004e-04	2.0399	2.118e-04	3.069e-03	0.58022	0.001	***
579841	1.416e-03	1.152e-03	1.2288	2.934e-03	1.887e-04	0.58188	0.002	**
837497	1.402e-03	7.796e-04	1.7986	2.866e-03	7.700e-05	0.58353	0.001	***
555348	1.397e-03	1.196e-03	1.1679	6.989e-05	2.862e-03	0.58517	0.001	***
2138725	1.389e-03	1.511e-03	0.9194	2.938e-03	2.988e-04	0.58680	0.001	***
593056	1.378e-03	9.014e-04	1.5291	3.948e-04	3.116e-03	0.58842	0.001	***
563843	1.375e-03	9.886e-04	1.3907	2.919e-03	2.318e-04	0.59003	0.436	
160817	1.374e-03	9.154e-04	1.5011	9.448e-04	3.503e-03	0.59164	1.000	
830429	1.372e-03	9.089e-04	1.5094	5.734e-04	3.208e-03	0.59325	0.001	***

```
137211                        1.363e-03 1.047e-03 1.3025 2.761e-03 3.907e-05 0.59486 0.001 ***
2188457                       1.356e-03 9.793e-04 1.3850 6.067e-04 3.282e-03 0.59645 0.001 ***
254226                        1.317e-03 1.295e-03 1.0171 2.701e-03 7.780e-05 0.59800 0.001 ***
4479015                       1.273e-03 8.822e-04 1.4427 1.161e-04 2.652e-03 0.59949 1.000
 [ reached getOption("max.print") -- omitted 7857 rows ]

Contrast: Bathypelagic_Surface

                                average        sd  ratio       ava       avb  cumsum     p
355538                        2.971e-02 2.629e-02 1.1300 1.169e-04 5.953e-02 0.03196 0.001 ***
557211                        1.978e-02 1.252e-02 1.5800 1.943e-03 4.150e-02 0.05324 0.001 ***
341618                        1.852e-02 5.984e-03 3.0942 8.314e-05 3.711e-02 0.07315 0.001 ***
639502                        1.784e-02 7.706e-03 2.3155 1.946e-04 3.588e-02 0.09235 0.001 ***
833419                        1.632e-02 7.459e-03 2.1880 3.325e-02 6.119e-04 0.10990 0.001 ***
226299                        1.603e-02 1.907e-02 0.8406 3.229e-02 2.474e-04 0.12714 0.001 ***
817600                        1.429e-02 1.106e-02 1.2924 5.665e-05 2.864e-02 0.14252 0.001 ***
630928                        1.214e-02 5.854e-03 2.0730 2.481e-02 5.465e-04 0.15557 0.001 ***
845691                        1.126e-02 5.650e-03 1.9937 2.304e-02 5.102e-04 0.16769 0.001 ***
228455                        1.086e-02 1.216e-02 0.8936 3.126e-05 2.176e-02 0.17938 0.001 ***
1080410                       1.059e-02 8.321e-03 1.2729 2.272e-02 1.865e-03 0.19077 1.000
839363                        1.012e-02 5.601e-03 1.8073 2.096e-02 7.233e-04 0.20166 0.002 **
427495                        9.719e-03 6.522e-03 1.4901 8.560e-04 2.029e-02 0.21211 0.001 ***
355527                        8.846e-03 8.215e-03 1.0769 4.734e-05 1.774e-02 0.22163 0.001 ***
1101488                       8.342e-03 2.099e-02 0.3975 1.779e-02 1.221e-03 0.23060 0.069 .
1082059                       8.196e-03 7.641e-03 1.0727 1.373e-02 1.195e-02 0.23942 0.001 ***
New.ReferenceOTU446           7.507e-03 1.842e-02 0.4076 1.526e-02 1.490e-03 0.24749 0.199
315563                        7.490e-03 1.141e-02 0.6565 3.564e-05 1.501e-02 0.25555 0.001 ***
1108577                       7.462e-03 3.791e-03 1.9686 8.373e-05 1.501e-02 0.26358 0.001 ***
884345                        6.690e-03 4.248e-03 1.5750 1.487e-02 1.926e-03 0.27077 0.976
592269                        6.232e-03 3.489e-03 1.7860 9.686e-05 1.256e-02 0.27748 0.001 ***
1138756                       6.151e-03 1.885e-02 0.3263 1.230e-02 5.513e-05 0.28409 0.108
351294                        5.730e-03 2.903e-03 1.9740 1.322e-02 1.923e-03 0.29026 0.001 ***
225284                        5.700e-03 6.660e-03 0.8558 1.148e-02 7.780e-05 0.29639 0.001 ***
1106009                       5.682e-03 2.513e-03 2.2609 1.585e-03 1.295e-02 0.30250 0.001 ***
1105428                       5.527e-03 5.271e-03 1.0485 6.344e-06 1.106e-02 0.30845 0.001 ***
```

1109816	5.358e-03	3.457e-03	1.5501	4.536e-05	1.076e-02	0.31421	0.001	***
New.ReferenceOTU882	5.229e-03	4.348e-03	1.2026	4.306e-05	1.050e-02	0.31983	0.001	***
1111501	5.202e-03	2.603e-03	1.9981	5.587e-05	1.046e-02	0.32543	0.001	***
160817	4.883e-03	3.037e-03	1.6080	1.013e-02	3.633e-04	0.33068	0.001	***
352531	4.846e-03	3.606e-03	1.3441	5.535e-06	9.698e-03	0.33590	0.001	***
554951	4.813e-03	2.057e-03	2.3395	9.786e-03	1.640e-04	0.34107	0.001	***
832816	4.801e-03	2.268e-03	2.1165	1.666e-04	9.769e-03	0.34624	0.001	***
592771	4.660e-03	2.800e-03	1.6642	2.209e-05	9.343e-03	0.35125	0.001	***
826517	4.618e-03	1.784e-03	2.5885	9.592e-03	3.585e-04	0.35622	0.001	***
353181	4.610e-03	2.379e-03	1.9375	3.941e-05	9.260e-03	0.36118	0.001	***
509913	4.598e-03	5.692e-03	0.8078	9.662e-03	4.726e-04	0.36612	0.001	***
4479015	4.522e-03	1.805e-03	2.5045	9.191e-03	1.475e-04	0.37099	0.001	***
141607	4.385e-03	4.913e-03	0.8926	9.380e-03	6.523e-04	0.37570	0.001	***
151344	4.146e-03	4.046e-03	1.0247	7.725e-05	8.360e-03	0.38016	0.001	***
341734	4.131e-03	2.685e-03	1.5385	1.469e-05	8.276e-03	0.38461	0.001	***
106179	4.114e-03	1.884e-03	2.1837	2.988e-05	8.257e-03	0.38903	0.001	***
343231	4.083e-03	3.546e-03	1.1516	3.533e-05	8.194e-03	0.39342	0.001	***
753560	4.028e-03	6.779e-03	0.5941	8.273e-03	2.445e-04	0.39776	0.017	*
231191	3.917e-03	4.144e-03	0.9454	3.995e-04	8.234e-03	0.40197	0.001	***
110119	3.821e-03	1.919e-03	1.9912	8.588e-03	1.083e-03	0.40608	0.001	***
338106	3.705e-03	5.323e-03	0.6961	8.527e-06	7.414e-03	0.41007	0.001	***
536001	3.689e-03	4.102e-03	0.8992	5.022e-04	7.657e-03	0.41404	0.001	***
227663	3.625e-03	3.352e-03	1.0812	1.531e-05	7.264e-03	0.41793	0.386	
358464	3.548e-03	9.700e-03	0.3657	7.086e-03	1.722e-05	0.42175	0.075	.
834169	3.382e-03	1.819e-03	1.8591	6.808e-03	4.418e-05	0.42539	0.001	***
3156804	3.365e-03	1.974e-03	1.7049	1.620e-04	6.879e-03	0.42901	0.001	***
New.ReferenceOTU128	3.328e-03	1.190e-03	2.7960	6.916e-03	2.603e-04	0.43259	0.001	***
1007856	3.234e-03	1.371e-03	2.3582	6.601e-03	1.401e-04	0.43607	0.001	***
310272	3.219e-03	2.368e-03	1.3595	6.705e-03	2.796e-04	0.43953	0.645	
323489	3.043e-03	3.049e-03	0.9980	6.612e-03	7.299e-04	0.44280	1.000	
711803	2.999e-03	3.963e-03	0.7566	6.006e-03	8.411e-06	0.44603	0.001	***
563843	2.878e-03	1.238e-03	2.3248	3.910e-05	5.795e-03	0.44912	0.001	***
1105105	2.851e-03	1.243e-03	2.2934	1.365e-04	5.839e-03	0.45219	0.001	***
646549	2.802e-03	3.751e-03	0.7470	6.182e-03	7.881e-04	0.45521	0.839	
551207	2.741e-03	3.011e-03	0.9104	6.036e-05	5.539e-03	0.45815	0.001	***

234927	2.726e-03	1.465e-03	1.8603	1.256e-04	5.574e-03	0.46109	0.001	***
581028	2.726e-03	1.706e-03	1.5979	5.717e-03	2.692e-04	0.46402	0.001	***
740763	2.696e-03	2.527e-03	1.0669	4.015e-06	5.397e-03	0.46692	0.001	***
844663	2.677e-03	1.598e-03	1.6753	5.465e-03	1.203e-04	0.46980	0.001	***
839203	2.675e-03	1.528e-03	1.7510	5.365e-03	1.516e-05	0.47268	0.001	***
542823	2.673e-03	1.300e-03	2.0562	5.139e-05	5.397e-03	0.47555	0.001	***
860929	2.569e-03	3.049e-03	0.8425	5.299e-03	2.123e-04	0.47831	0.001	***
New.ReferenceOTU44	2.558e-03	2.118e-03	1.2078	5.494e-03	3.896e-04	0.48106	0.495	
592350	2.540e-03	2.415e-03	1.0518	2.453e-05	5.104e-03	0.48380	0.001	***
591130	2.495e-03	2.577e-03	0.9683	1.517e-04	5.109e-03	0.48648	0.001	***
842800	2.474e-03	1.529e-03	1.6186	5.150e-03	2.605e-04	0.48914	0.973	
777466	2.431e-03	3.333e-03	0.7295	5.274e-03	1.433e-03	0.49176	0.987	
234994	2.397e-03	2.323e-03	1.0317	8.137e-06	4.802e-03	0.49434	0.001	***
773487	2.379e-03	1.363e-03	1.7455	4.273e-05	4.799e-03	0.49689	0.001	***
979107	2.329e-03	4.290e-03	0.5429	4.800e-03	1.963e-04	0.49940	0.037	*
832702	2.273e-03	8.449e-04	2.6899	4.619e-03	7.485e-05	0.50184	0.001	***
232905	2.268e-03	1.117e-03	2.0299	3.988e-05	4.573e-03	0.50428	0.001	***
774258	2.203e-03	2.898e-03	0.7601	4.456e-04	4.616e-03	0.50665	0.001	***
1107076	2.181e-03	4.508e-03	0.4838	4.118e-03	8.876e-04	0.50900	0.709	
834856	2.162e-03	1.260e-03	1.7157	4.546e-03	2.394e-04	0.51132	0.001	***
314474	2.160e-03	1.247e-03	1.7322	1.297e-06	4.322e-03	0.51365	0.001	***
249226	2.146e-03	1.014e-03	2.1171	1.462e-06	4.293e-03	0.51596	0.001	***
832374	2.124e-03	9.182e-04	2.3131	4.411e-03	1.809e-04	0.51824	0.001	***
New.ReferenceOTU697	2.115e-03	7.445e-04	2.8411	4.275e-03	4.448e-05	0.52052	0.001	***
New.ReferenceOTU238	2.076e-03	1.076e-03	1.9301	2.631e-04	4.406e-03	0.52275	0.001	***
846727	2.056e-03	8.294e-04	2.4785	4.433e-03	3.506e-04	0.52496	0.001	***
810040	2.050e-03	9.258e-04	2.2144	4.125e-03	2.497e-05	0.52717	0.001	***
4475220	2.028e-03	1.623e-03	1.2494	9.714e-06	4.066e-03	0.52935	0.001	***
1107422	2.012e-03	3.019e-03	0.6663	1.959e-07	4.023e-03	0.53151	0.001	***
New.ReferenceOTU252	1.994e-03	3.035e-03	0.6569	0.000e+00	3.987e-03	0.53366	0.001	***
227866	1.986e-03	2.015e-03	0.9857	1.304e-05	3.985e-03	0.53579	0.001	***
539147	1.974e-03	3.013e-03	0.6551	1.890e-05	3.961e-03	0.53792	0.963	
New.ReferenceOTU414	1.947e-03	2.014e-03	0.9671	4.462e-03	8.273e-04	0.54001	0.001	***
New.ReferenceOTU399	1.947e-03	8.042e-04	2.4206	2.384e-04	4.131e-03	0.54211	0.001	***
356368	1.934e-03	9.331e-04	2.0726	9.767e-06	3.878e-03	0.54419	0.001	***

```
830634                        1.921e-03 1.312e-03 1.4649 3.931e-03 1.159e-04 0.54625 0.002 **
544313                        1.901e-03 2.294e-03 0.8288 3.896e-03 1.185e-04 0.54830 0.001 ***
569884                        1.900e-03 1.510e-03 1.2580 1.509e-05 3.815e-03 0.55034 0.001 ***
838079                        1.889e-03 1.015e-03 1.8616 3.787e-03 9.425e-06 0.55237 0.001 ***
548576                        1.868e-03 3.552e-03 0.5260 3.759e-03 6.854e-05 0.55438 0.009 **
1011088                       1.852e-03 2.683e-03 0.6904 1.824e-03 3.224e-03 0.55637 0.001 ***
1084865                       1.838e-03 1.890e-03 0.9728 3.979e-03 3.225e-04 0.55835 0.001 ***
836341                        1.832e-03 1.005e-03 1.8234 3.798e-03 1.366e-04 0.56032 0.001 ***
New.ReferenceOTU698           1.824e-03 1.096e-03 1.6646 2.000e-05 3.668e-03 0.56228 0.001 ***
344865                        1.817e-03 8.415e-04 2.1586 9.963e-05 3.706e-03 0.56424 0.001 ***
347913                        1.805e-03 1.144e-03 1.5773 3.779e-03 2.107e-04 0.56618 0.321
834104                        1.801e-03 8.841e-04 2.0375 3.732e-03 1.405e-04 0.56812 0.001 ***
182418                        1.797e-03 3.957e-03 0.4542 3.626e-03 1.113e-04 0.57005 0.049 *
243160                        1.793e-03 1.746e-03 1.0269 1.101e-05 3.596e-03 0.57198 0.001 ***
1109402                       1.789e-03 1.423e-03 1.2568 7.672e-06 3.585e-03 0.57390 0.001 ***
833273                        1.787e-03 9.082e-04 1.9676 3.657e-03 8.430e-05 0.57583 0.001 ***
579841                        1.773e-03 1.273e-03 1.3921 3.447e-06 3.549e-03 0.57773 0.001 ***
827407                        1.772e-03 8.938e-04 1.9820 3.790e-03 2.704e-04 0.57964 0.001 ***
108953                        1.757e-03 8.166e-04 2.1511 3.629e-03 1.219e-04 0.58153 0.001 ***
826571                        1.748e-03 7.713e-04 2.2669 3.522e-03 2.501e-05 0.58341 0.001 ***
841316                        1.741e-03 8.070e-04 2.1573 3.602e-03 1.264e-04 0.58528 0.001 ***
583907                        1.738e-03 4.467e-03 0.3891 3.467e-03 3.168e-05 0.58715 0.073 .
103709                        1.737e-03 8.418e-04 2.0630 2.347e-05 3.497e-03 0.58902 0.001 ***
227717                        1.734e-03 7.979e-04 2.1728 2.473e-05 3.492e-03 0.59088 0.001 ***
779725                        1.723e-03 1.005e-03 1.7138 3.613e-03 1.714e-04 0.59274 0.001 ***
1108696                       1.712e-03 1.272e-03 1.3454 1.551e-03 3.868e-03 0.59458 0.001 ***
112306                        1.680e-03 9.740e-04 1.7253 3.408e-03 5.439e-05 0.59639 0.001 ***
757981                        1.678e-03 9.431e-04 1.7789 3.382e-03 2.772e-05 0.59819 0.001 ***
926160                        1.675e-03 8.624e-03 0.1943 3.274e-03 1.245e-04 0.59999 0.325
 [ reached getOption("max.print") -- omitted 7857 rows ]

Contrast: Bathypelagic_Mesopelagic

                                average        sd  ratio       ava       avb  cumsum     p
1080410                       2.555e-02 1.625e-02 1.5723 2.272e-02 7.157e-02 0.04170 0.001 ***
```

323489	1.709e-02	1.311e-02	1.3036	6.612e-03	4.023e-02	0.06959	0.001	***
34469	1.577e-02	7.799e-03	2.0218	2.774e-03	3.431e-02	0.09533	0.001	***
226299	1.537e-02	1.901e-02	0.8086	3.229e-02	1.765e-03	0.12041	0.001	***
884345	9.498e-03	6.841e-03	1.3885	1.487e-02	3.115e-02	0.13591	0.001	***
New.ReferenceOTU446	9.024e-03	1.797e-02	0.5022	1.526e-02	7.663e-03	0.15064	0.003	**
833419	8.381e-03	6.698e-03	1.2512	3.325e-02	1.999e-02	0.16432	0.899	
1101488	8.300e-03	2.064e-02	0.4021	1.779e-02	3.782e-03	0.17787	0.020	*
839363	7.906e-03	6.309e-03	1.2533	2.096e-02	3.017e-02	0.19077	0.911	
630928	6.659e-03	5.388e-03	1.2358	2.481e-02	1.414e-02	0.20164	0.648	
845691	6.430e-03	4.801e-03	1.3394	2.304e-02	1.560e-02	0.21213	0.777	
1138756	6.352e-03	1.869e-02	0.3399	1.230e-02	7.556e-04	0.22250	0.023	*
108965	6.150e-03	3.513e-03	1.7504	1.332e-03	1.361e-02	0.23254	0.001	***
225284	5.413e-03	6.620e-03	0.8177	1.148e-02	8.007e-04	0.24137	0.001	***
646549	4.692e-03	6.192e-03	0.7577	6.182e-03	9.228e-03	0.24903	0.003	**
1082059	4.335e-03	3.268e-03	1.3268	1.373e-02	6.702e-03	0.25610	0.946	
509913	4.099e-03	5.513e-03	0.7435	9.662e-03	2.193e-03	0.26279	0.001	***
141607	4.050e-03	4.802e-03	0.8434	9.380e-03	2.648e-03	0.26940	0.002	**
753560	4.048e-03	6.365e-03	0.6360	8.273e-03	3.340e-03	0.27601	0.003	**
842800	3.681e-03	2.859e-03	1.2874	5.150e-03	1.146e-02	0.28202	0.001	***
358464	3.678e-03	9.595e-03	0.3833	7.086e-03	3.925e-04	0.28802	0.012	*
351294	3.677e-03	2.799e-03	1.3139	1.322e-02	1.762e-02	0.29402	1.000	
310272	3.660e-03	2.555e-03	1.4326	6.705e-03	1.186e-02	0.30000	0.032	*
777466	3.654e-03	4.454e-03	0.8203	5.274e-03	7.329e-03	0.30596	0.945	
160817	3.462e-03	3.004e-03	1.1524	1.013e-02	3.503e-03	0.31161	0.001	***
1107076	3.427e-03	3.568e-03	0.9606	4.118e-03	5.312e-03	0.31721	0.001	***
New.ReferenceOTU44	3.391e-03	5.147e-03	0.6588	5.494e-03	6.929e-03	0.32274	0.013	*
4479015	3.343e-03	1.869e-03	1.7880	9.191e-03	2.652e-03	0.32820	0.001	***
834169	3.068e-03	1.853e-03	1.6556	6.808e-03	7.042e-04	0.33320	0.001	***
711803	2.983e-03	3.963e-03	0.7527	6.006e-03	3.929e-05	0.33807	0.001	***
826533	2.889e-03	5.875e-03	0.4918	1.710e-03	4.987e-03	0.34279	0.004	**
554951	2.886e-03	1.790e-03	1.6117	9.786e-03	4.902e-03	0.34750	0.011	*
315098	2.775e-03	1.662e-03	1.6695	1.726e-04	5.723e-03	0.35203	0.003	**
573845	2.759e-03	4.835e-03	0.5707	1.794e-03	4.528e-03	0.35653	0.002	**
New.ReferenceOTU128	2.535e-03	1.239e-03	2.0457	6.916e-03	1.868e-03	0.36067	0.001	***
839203	2.531e-03	1.538e-03	1.6457	5.365e-03	3.051e-04	0.36480	0.001	***

837317	2.527e-03	2.025e-03	1.2477	2.500e-03	7.094e-03	0.36893	0.001	***
125125	2.496e-03	1.408e-03	1.7726	2.803e-03	7.662e-03	0.37300	0.001	***
860929	2.463e-03	2.975e-03	0.8279	5.299e-03	6.726e-04	0.37702	0.001	***
154513	2.459e-03	2.029e-03	1.2119	9.039e-04	5.746e-03	0.38103	0.001	***
979107	2.420e-03	4.077e-03	0.5934	4.800e-03	1.909e-03	0.38498	0.001	***
831163	2.255e-03	1.411e-03	1.5979	4.451e-04	4.951e-03	0.38866	0.001	***
844663	2.248e-03	1.508e-03	1.4910	5.465e-03	1.195e-03	0.39233	0.001	***
253327	2.216e-03	3.182e-03	0.6963	4.464e-04	4.478e-03	0.39595	0.001	***
325965	2.163e-03	1.318e-03	1.6405	2.118e-03	6.220e-03	0.39948	0.001	***
6258	2.162e-03	1.064e-03	2.0317	1.485e-06	4.325e-03	0.40301	0.001	***
826517	2.019e-03	1.546e-03	1.3057	9.592e-03	7.403e-03	0.40630	1.000	
347857	1.954e-03	9.238e-04	2.1151	4.328e-04	4.310e-03	0.40949	0.001	***
355538	1.907e-03	3.247e-03	0.5875	1.169e-04	3.879e-03	0.41261	1.000	
837714	1.878e-03	9.646e-04	1.9465	2.098e-03	5.786e-03	0.41567	0.001	***
583907	1.875e-03	4.346e-03	0.4315	3.467e-03	4.776e-04	0.41873	0.006	**
544313	1.863e-03	2.223e-03	0.8384	3.896e-03	9.077e-04	0.42177	0.001	***
182418	1.853e-03	3.817e-03	0.4855	3.626e-03	8.281e-04	0.42480	0.003	**
110119	1.850e-03	1.418e-03	1.3045	8.588e-03	8.296e-03	0.42782	1.000	
548576	1.849e-03	3.465e-03	0.5336	3.759e-03	5.187e-04	0.43083	0.001	***
810040	1.815e-03	9.622e-04	1.8861	4.125e-03	5.172e-04	0.43380	0.001	***
New.ReferenceOTU414	1.803e-03	1.981e-03	0.9103	4.462e-03	1.182e-03	0.43674	0.001	***
838079	1.775e-03	1.015e-03	1.7487	3.787e-03	2.508e-04	0.43964	0.001	***
830634	1.768e-03	1.498e-03	1.1802	3.931e-03	3.309e-03	0.44252	0.005	**
926160	1.752e-03	8.591e-03	0.2040	3.274e-03	3.758e-04	0.44538	0.145	
347913	1.750e-03	1.252e-03	1.3978	3.779e-03	6.481e-03	0.44824	0.488	
New.ReferenceOTU697	1.732e-03	8.293e-04	2.0880	4.275e-03	9.513e-04	0.45106	0.001	***
New.ReferenceOTU752	1.692e-03	4.784e-03	0.3537	3.229e-03	9.303e-04	0.45383	0.013	*
956811	1.640e-03	2.217e-03	0.7401	2.991e-03	2.568e-03	0.45650	0.940	
557211	1.624e-03	2.075e-03	0.7824	1.943e-03	3.492e-03	0.45915	1.000	
161219	1.611e-03	8.233e-04	1.9568	5.035e-04	3.692e-03	0.46178	0.001	***
552671	1.588e-03	2.192e-03	0.7246	3.380e-03	3.395e-04	0.46438	0.001	***
1074804	1.571e-03	7.838e-04	2.0037	2.226e-04	3.362e-03	0.46694	0.001	***
581028	1.561e-03	1.496e-03	1.0429	5.717e-03	3.866e-03	0.46949	0.721	
1084865	1.543e-03	1.716e-03	0.8992	3.979e-03	1.987e-03	0.47200	0.001	***
825733	1.513e-03	9.170e-04	1.6504	2.119e-03	4.945e-03	0.47447	0.001	***

101670	1.488e-03	9.077e-04	1.6398	1.031e-03	3.872e-03	0.47690	0.001	***
825985	1.479e-03	1.015e-03	1.4577	2.469e-03	5.150e-03	0.47932	0.003	**
834243	1.473e-03	1.508e-03	0.9772	1.243e-03	3.398e-03	0.48172	0.001	***
1007856	1.469e-03	1.113e-03	1.3190	6.601e-03	4.783e-03	0.48412	1.000	
757981	1.447e-03	9.389e-04	1.5406	3.382e-03	5.319e-04	0.48648	0.001	***
404788	1.442e-03	2.621e-03	0.5503	2.662e-03	9.214e-04	0.48883	0.015	*
826571	1.439e-03	7.993e-04	1.8001	3.522e-03	6.593e-04	0.49118	0.001	***
834856	1.402e-03	1.004e-03	1.3956	4.546e-03	5.917e-03	0.49347	1.000	
832702	1.388e-03	8.493e-04	1.6337	4.619e-03	1.983e-03	0.49573	0.001	***
2188457	1.362e-03	9.824e-04	1.3860	5.916e-04	3.282e-03	0.49796	0.001	***
639502	1.349e-03	2.098e-03	0.6430	1.946e-04	2.811e-03	0.50016	1.000	
631576	1.333e-03	1.933e-03	0.6899	1.875e-03	1.763e-03	0.50233	0.002	**
837290	1.313e-03	7.775e-04	1.6893	3.028e-03	4.114e-04	0.50448	0.001	***
824150	1.309e-03	1.976e-03	0.6624	2.666e-03	9.795e-04	0.50662	0.001	***
1128468	1.309e-03	2.101e-03	0.6228	2.136e-03	1.317e-03	0.50875	0.937	
278985	1.283e-03	1.232e-03	1.0417	2.592e-03	2.086e-03	0.51085	0.001	***
832374	1.270e-03	9.513e-04	1.3353	4.411e-03	6.062e-03	0.51292	1.000	
779725	1.240e-03	9.455e-04	1.3112	3.613e-03	1.461e-03	0.51494	0.001	***
836341	1.229e-03	1.008e-03	1.2189	3.798e-03	1.574e-03	0.51695	0.006	**
112306	1.220e-03	8.640e-04	1.4125	3.408e-03	1.372e-03	0.51894	0.001	***
794237	1.195e-03	5.873e-04	2.0344	2.595e-03	2.160e-04	0.52089	0.001	***
833273	1.184e-03	8.951e-04	1.3224	3.657e-03	1.432e-03	0.52282	0.001	***
739517	1.177e-03	6.140e-04	1.9170	2.733e-03	4.111e-04	0.52474	0.001	***
833556	1.171e-03	8.024e-04	1.4588	2.788e-03	6.371e-04	0.52665	0.001	***
New.ReferenceOTU900	1.169e-03	3.708e-03	0.3153	2.202e-03	4.304e-04	0.52856	0.002	**
846727	1.149e-03	9.123e-04	1.2599	4.433e-03	4.824e-03	0.53044	1.000	
827407	1.134e-03	8.405e-04	1.3495	3.790e-03	5.217e-03	0.53229	1.000	
1108205	1.107e-03	2.574e-03	0.4298	2.237e-03	3.004e-04	0.53410	0.002	**
New.ReferenceOTU421	1.102e-03	7.517e-04	1.4659	3.068e-03	4.881e-03	0.53589	0.998	
822991	1.096e-03	1.693e-03	0.6474	1.372e-03	1.188e-03	0.53768	0.005	**
834796	1.074e-03	1.153e-03	0.9310	3.623e-06	2.151e-03	0.53944	0.001	***
830429	1.066e-03	8.434e-04	1.2636	1.469e-03	3.208e-03	0.54118	0.001	***
838996	1.058e-03	8.344e-04	1.2676	1.006e-03	3.061e-03	0.54290	0.001	***
555348	1.053e-03	1.043e-03	1.0090	1.332e-03	2.862e-03	0.54462	0.001	***
New.ReferenceOTU219	1.052e-03	7.011e-04	1.5011	3.758e-04	2.435e-03	0.54634	0.001	***

```
834286                      1.039e-03 8.648e-04 1.2013 2.521e-03 3.720e-03 0.54803 0.736
838426                      9.714e-04 8.708e-04 1.1155 2.867e-03 1.083e-03 0.54962 0.001 ***
344394                      9.690e-04 1.932e-03 0.5017 6.130e-06 1.939e-03 0.55120 0.003 **
593056                      9.457e-04 7.019e-04 1.3473 2.741e-03 3.116e-03 0.55274 0.878
108953                      9.412e-04 6.764e-04 1.3915 3.629e-03 2.447e-03 0.55428 0.956
834104                      9.337e-04 6.998e-04 1.3341 3.732e-03 3.069e-03 0.55580 1.000
841316                      9.271e-04 7.002e-04 1.3240 3.602e-03 2.122e-03 0.55732 0.631
948029                      9.202e-04 9.731e-04 0.9457 1.938e-03 8.273e-04 0.55882 0.073 .
1660169                     9.174e-04 7.856e-04 1.1677 2.593e-03 1.215e-03 0.56032 0.003 **
New.ReferenceOTU521         9.088e-04 6.251e-04 1.4539 2.499e-03 1.141e-03 0.56180 0.001 ***
833549                      9.064e-04 5.469e-04 1.6573 1.846e-03 3.735e-05 0.56328 0.001 ***
New.ReferenceOTU305         8.977e-04 5.713e-04 1.5713 2.908e-04 2.067e-03 0.56474 0.001 ***
560276                      8.905e-04 5.765e-04 1.5448 1.797e-04 1.953e-03 0.56620 1.000
309877                      8.708e-04 1.080e-03 0.8060 1.995e-03 6.582e-04 0.56762 0.019 *
1127320                     8.686e-04 6.625e-04 1.3112 3.928e-05 1.769e-03 0.56904 0.001 ***
829681                      8.649e-04 4.348e-04 1.9894 4.727e-05 1.773e-03 0.57045 0.001 ***
New.ReferenceOTU17          8.629e-04 8.994e-04 0.9594 6.228e-05 1.776e-03 0.57186 0.001 ***
1108696                     8.554e-04 1.027e-03 0.8329 1.551e-03 1.046e-03 0.57325 0.990
1011088                     8.525e-04 2.553e-03 0.3340 1.824e-03 3.677e-04 0.57464 0.789
 [ reached getOption("max.print") -- omitted 7857 rows ]

Contrast: Surface_Mesopelagic

                              average        sd  ratio       ava       avb  cumsum     p
1080410                     3.485e-02 1.594e-02 2.1868 1.865e-03 7.157e-02 0.03879 0.001 ***
355538                      2.819e-02 2.611e-02 1.0798 5.953e-02 3.879e-03 0.07017 0.002 **
323489                      1.975e-02 1.313e-02 1.5045 7.299e-04 4.023e-02 0.09215 0.001 ***
557211                      1.911e-02 1.248e-02 1.5309 4.150e-02 3.492e-03 0.11342 0.001 ***
341618                      1.802e-02 6.029e-03 2.9880 3.711e-02 1.082e-03 0.13347 0.001 ***
34469                       1.681e-02 7.762e-03 2.1662 6.814e-04 3.431e-02 0.15219 0.001 ***
639502                      1.654e-02 7.989e-03 2.0698 3.588e-02 2.811e-03 0.17059 0.001 ***
839363                      1.472e-02 7.156e-03 2.0576 7.233e-04 3.017e-02 0.18698 0.001 ***
884345                      1.467e-02 7.408e-03 1.9800 1.926e-03 3.115e-02 0.20330 0.001 ***
817600                      1.418e-02 1.106e-02 1.2819 2.864e-02 2.826e-04 0.21909 0.001 ***
228455                      1.072e-02 1.216e-02 0.8813 2.176e-02 3.306e-04 0.23101 0.001 ***
```

833419	9.695e-03	4.105e-03	2.3618	6.119e-04	1.999e-02	0.24180	0.155	
427495	9.534e-03	6.531e-03	1.4598	2.029e-02	1.237e-03	0.25242	0.001	***
355527	8.787e-03	8.217e-03	1.0694	1.774e-02	1.656e-04	0.26220	0.001	***
351294	7.867e-03	3.133e-03	2.5109	1.923e-03	1.762e-02	0.27095	0.001	***
845691	7.544e-03	4.388e-03	1.7192	5.102e-04	1.560e-02	0.27935	0.041	*
315563	7.486e-03	1.141e-02	0.6562	1.501e-02	4.518e-05	0.28768	0.001	***
1108577	7.396e-03	3.793e-03	1.9501	1.501e-02	2.150e-04	0.29591	0.001	***
630928	6.824e-03	3.410e-03	2.0012	5.465e-04	1.414e-02	0.30351	0.458	
108965	6.649e-03	3.471e-03	1.9154	3.123e-04	1.361e-02	0.31091	0.001	***
1082059	6.266e-03	9.018e-03	0.6949	1.195e-02	6.702e-03	0.31788	0.136	
592269	6.179e-03	3.498e-03	1.7666	1.256e-02	2.179e-04	0.32476	0.001	***
310272	5.790e-03	2.789e-03	2.0763	2.796e-04	1.186e-02	0.33120	0.001	***
842800	5.606e-03	3.091e-03	1.8138	2.605e-04	1.146e-02	0.33744	0.001	***
1105428	5.445e-03	5.263e-03	1.0346	1.106e-02	2.092e-04	0.34350	0.001	***
1109816	5.155e-03	3.454e-03	1.4927	1.076e-02	5.120e-04	0.34924	0.001	***
New.ReferenceOTU882	5.131e-03	4.353e-03	1.1787	1.050e-02	2.413e-04	0.35495	0.001	***
1106009	5.029e-03	2.580e-03	1.9493	1.295e-02	2.894e-03	0.36055	0.001	***
1111501	4.777e-03	2.675e-03	1.7861	1.046e-02	9.192e-04	0.36586	0.001	***
352531	4.774e-03	3.608e-03	1.3234	9.698e-03	1.493e-04	0.37118	0.001	***
592771	4.606e-03	2.805e-03	1.6416	9.343e-03	1.320e-04	0.37630	0.001	***
832816	4.536e-03	2.303e-03	1.9699	9.769e-03	7.112e-04	0.38135	0.001	***
353181	4.413e-03	2.413e-03	1.8285	9.260e-03	4.774e-04	0.38626	0.001	***
646549	4.405e-03	6.525e-03	0.6752	7.881e-04	9.228e-03	0.39117	0.075	.
151344	4.151e-03	4.047e-03	1.0257	8.360e-03	6.489e-05	0.39579	0.001	***
341734	4.115e-03	2.686e-03	1.5324	8.276e-03	4.504e-05	0.40037	0.001	***
106179	4.051e-03	1.887e-03	2.1463	8.257e-03	1.558e-04	0.40487	0.001	***
343231	4.019e-03	3.502e-03	1.1478	8.194e-03	3.035e-04	0.40935	0.001	***
125125	3.704e-03	1.373e-03	2.6985	2.566e-04	7.662e-03	0.41347	0.001	***
338106	3.701e-03	5.300e-03	0.6983	7.414e-03	9.064e-05	0.41759	0.001	***
536001	3.646e-03	4.035e-03	0.9036	7.657e-03	7.741e-04	0.42165	0.001	***
110119	3.644e-03	1.666e-03	2.1866	1.083e-03	8.296e-03	0.42570	0.001	***
New.ReferenceOTU446	3.609e-03	6.381e-03	0.5657	1.490e-03	7.663e-03	0.42972	0.898	
826517	3.527e-03	1.544e-03	2.2836	3.585e-04	7.403e-03	0.43365	0.001	***
231191	3.524e-03	4.144e-03	0.8502	8.234e-03	1.200e-03	0.43757	0.017	*
837317	3.424e-03	2.075e-03	1.6500	3.439e-04	7.094e-03	0.44138	0.001	***

227663	3.354e-03	3.266e-03	1.0269	7.264e-03	1.079e-03	0.44511	0.626	
777466	3.303e-03	4.330e-03	0.7629	1.433e-03	7.329e-03	0.44879	0.902	
New.ReferenceOTU44	3.287e-03	5.744e-03	0.5723	3.896e-04	6.929e-03	0.45245	0.095	.
3156804	3.235e-03	1.978e-03	1.6352	6.879e-03	4.141e-04	0.45605	0.001	***
347913	3.136e-03	1.259e-03	2.4906	2.107e-04	6.481e-03	0.45954	0.001	***
325965	3.025e-03	1.281e-03	2.3604	1.710e-04	6.220e-03	0.46290	0.001	***
832374	2.944e-03	1.030e-03	2.8574	1.809e-04	6.062e-03	0.46618	0.001	***
834856	2.839e-03	9.894e-04	2.8690	2.394e-04	5.917e-03	0.46934	0.001	***
563843	2.782e-03	1.264e-03	2.2004	5.795e-03	2.318e-04	0.47244	0.001	***
837714	2.774e-03	8.507e-04	3.2604	2.389e-04	5.786e-03	0.47552	0.001	***
154513	2.773e-03	2.046e-03	1.3553	2.065e-04	5.746e-03	0.47861	0.001	***
1105105	2.759e-03	1.254e-03	2.2002	5.839e-03	3.208e-04	0.48168	0.001	***
234927	2.706e-03	1.465e-03	1.8473	5.574e-03	1.729e-04	0.48469	0.001	***
551207	2.678e-03	3.002e-03	0.8918	5.539e-03	2.209e-04	0.48767	0.001	***
740763	2.672e-03	2.526e-03	1.0578	5.397e-03	5.884e-05	0.49065	0.001	***
315098	2.525e-03	1.623e-03	1.5555	9.621e-04	5.723e-03	0.49346	0.102	
542823	2.505e-03	1.325e-03	1.8911	5.397e-03	3.862e-04	0.49624	0.001	***
591130	2.505e-03	2.584e-03	0.9692	5.109e-03	1.051e-04	0.49903	0.001	***
825985	2.480e-03	1.015e-03	2.4426	1.946e-04	5.150e-03	0.50179	0.001	***
827407	2.474e-03	8.815e-04	2.8061	2.704e-04	5.217e-03	0.50454	0.001	***
826533	2.459e-03	5.872e-03	0.4188	2.813e-04	4.987e-03	0.50728	0.144	
592350	2.424e-03	2.415e-03	1.0034	5.104e-03	2.691e-04	0.50998	0.001	***
573845	2.410e-03	4.844e-03	0.4975	5.011e-04	4.528e-03	0.51266	0.084	.
1107076	2.410e-03	1.562e-03	1.5426	8.876e-04	5.312e-03	0.51534	0.486	
773487	2.394e-03	1.363e-03	1.7558	4.799e-03	1.128e-05	0.51801	0.001	***
234994	2.379e-03	2.318e-03	1.0264	4.802e-03	5.808e-05	0.52065	0.001	***
554951	2.373e-03	1.196e-03	1.9839	1.640e-04	4.902e-03	0.52330	0.895	
831163	2.359e-03	1.407e-03	1.6762	2.362e-04	4.951e-03	0.52592	0.001	***
New.ReferenceOTU421	2.340e-03	7.350e-04	3.1832	2.038e-04	4.881e-03	0.52853	0.001	***
1007856	2.325e-03	8.339e-04	2.7883	1.401e-04	4.783e-03	0.53111	0.001	***
825733	2.314e-03	9.434e-04	2.4531	3.288e-04	4.945e-03	0.53369	0.001	***
232905	2.262e-03	1.118e-03	2.0236	4.573e-03	5.023e-05	0.53621	0.001	***
846727	2.256e-03	1.249e-03	1.8058	3.506e-04	4.824e-03	0.53872	0.001	***
774258	2.207e-03	2.851e-03	0.7741	4.616e-03	7.058e-04	0.54117	0.001	***
253327	2.192e-03	3.039e-03	0.7214	1.397e-03	4.478e-03	0.54361	0.002	**

314474	2.148e-03	1.247e-03	1.7215	4.322e-03	2.708e-05	0.54600	0.001	***
New.ReferenceOTU238	2.123e-03	1.064e-03	1.9947	4.406e-03	1.645e-04	0.54837	0.001	***
347857	2.108e-03	9.363e-04	2.2511	1.001e-04	4.310e-03	0.55071	0.001	***
249226	2.092e-03	1.023e-03	2.0449	4.293e-03	1.104e-04	0.55304	0.001	***
1107422	2.010e-03	3.016e-03	0.6666	4.023e-03	1.282e-05	0.55528	0.001	***
4475220	1.996e-03	1.624e-03	1.2288	4.066e-03	7.846e-05	0.55750	0.001	***
New.ReferenceOTU252	1.994e-03	3.033e-03	0.6574	3.987e-03	5.910e-06	0.55972	0.001	***
6258	1.989e-03	1.070e-03	1.8583	3.601e-04	4.325e-03	0.56193	0.001	***
227866	1.959e-03	2.006e-03	0.9765	3.985e-03	9.696e-05	0.56411	0.001	***
356368	1.934e-03	9.331e-04	2.0724	3.878e-03	1.038e-05	0.56626	0.001	***
New.ReferenceOTU399	1.899e-03	8.068e-04	2.3541	4.131e-03	3.350e-04	0.56838	0.001	***
539147	1.880e-03	2.900e-03	0.6482	3.961e-03	8.619e-04	0.57047	0.982	
101670	1.876e-03	9.348e-04	2.0070	1.281e-04	3.872e-03	0.57256	0.001	***
569884	1.871e-03	1.511e-03	1.2384	3.815e-03	7.257e-05	0.57464	0.001	***
834286	1.808e-03	9.440e-04	1.9152	1.062e-04	3.720e-03	0.57665	0.001	***
581028	1.804e-03	9.857e-04	1.8298	2.692e-04	3.866e-03	0.57866	0.128	
161219	1.787e-03	8.223e-04	2.1732	1.248e-04	3.692e-03	0.58065	0.001	***
1109402	1.786e-03	1.423e-03	1.2554	3.585e-03	1.455e-05	0.58264	0.001	***
243160	1.779e-03	1.734e-03	1.0262	3.596e-03	8.884e-05	0.58462	0.001	***
344865	1.779e-03	8.457e-04	2.1036	3.706e-03	1.877e-04	0.58660	0.001	***
New.ReferenceOTU698	1.720e-03	1.098e-03	1.5657	3.668e-03	2.650e-04	0.58851	0.005	**
579841	1.710e-03	1.260e-03	1.3575	3.549e-03	1.887e-04	0.59042	0.001	***
119137	1.686e-03	1.457e-03	1.1577	3.876e-03	5.858e-04	0.59229	0.052	.
834243	1.661e-03	1.724e-03	0.9632	1.134e-04	3.398e-03	0.59414	0.001	***
830634	1.631e-03	1.873e-03	0.8710	1.159e-04	3.309e-03	0.59596	0.150	
1074804	1.631e-03	7.754e-04	2.1030	1.008e-04	3.362e-03	0.59777	0.001	***
323305	1.630e-03	2.324e-03	0.7013	3.273e-03	1.401e-05	0.59958	0.001	***
227717	1.614e-03	8.327e-04	1.9378	3.492e-03	2.750e-04	0.60138	0.001	***
344629	1.607e-03	1.576e-03	1.0198	3.378e-03	3.624e-04	0.60317	0.001	***
160817	1.593e-03	9.416e-04	1.6924	3.633e-04	3.503e-03	0.60494	1.000	
103709	1.582e-03	8.505e-04	1.8596	3.497e-03	4.817e-04	0.60670	0.001	***
753560	1.571e-03	2.170e-03	0.7239	2.445e-04	3.340e-03	0.60845	0.957	
2188457	1.569e-03	9.800e-04	1.6008	1.445e-04	3.282e-03	0.61020	0.001	***
354725	1.557e-03	6.018e-04	2.5869	3.170e-03	5.867e-05	0.61193	0.001	***
1108696	1.556e-03	1.232e-03	1.2628	3.868e-03	1.046e-03	0.61366	0.001	***

```
830429                          1.542e-03 9.113e-04 1.6920 1.327e-04 3.208e-03 0.61538 0.001 ***
229118                          1.519e-03 8.174e-04 1.8589 3.207e-03 1.692e-04 0.61707 0.001 ***
593056                          1.508e-03 8.982e-04 1.6786 1.123e-04 3.116e-03 0.61875 0.001 ***
147786                          1.505e-03 1.627e-03 0.9246 3.066e-03 1.165e-04 0.62042 0.001 ***
1011088                         1.504e-03 1.811e-03 0.8306 3.224e-03 3.677e-04 0.62209 0.062 .
1101488                         1.503e-03 2.086e-03 0.7206 1.221e-03 3.782e-03 0.62377 0.986
838996                          1.496e-03 8.424e-04 1.7753 8.678e-05 3.061e-03 0.62543 0.001 ***
834104                          1.468e-03 7.058e-04 2.0796 1.405e-04 3.069e-03 0.62707 0.001 ***
546944                          1.461e-03 7.533e-04 1.9389 2.941e-03 1.961e-05 0.62869 0.001 ***
 [ reached getOption("max.print") -- omitted 7857 rows ]
---
Signif. codes:  0 ‘***’ 0.001 ‘**’ 0.01 ‘*’ 0.05 ‘.’ 0.1 ‘ ’ 1
Permutation: free
Number of permutations: 999
```
